# Transcriptome-guided metabolic network analysis reveals rearrangements of carbon flux distribution in *Neisseria gonorrhoeae* during neutrophil co-culture

**DOI:** 10.1128/msystems.01265-22

**Published:** 2023-06-30

**Authors:** Aimee D. Potter, Christopher M. Baiocco, Jason A. Papin, Alison K. Criss

**Affiliations:** 1 Department of Microbiology, Immunology, and Cancer Biology, University of Virginia, Charlottesville, Virginia, USA; 2 Department of Biomedical Engineering, University of Virginia, Charlottesville, Virginia, USA; Mayo Clinic, Rochester, Minnesota, USA

**Keywords:** metabolism, *Neisseria gonorrhoeae*, genome-scale metabolic network reconstruction, transcriptomics, neutrophils, metabolic network analysis, carbon metabolism

## Abstract

The ability of bacterial pathogens to metabolically adapt to the environmental conditions of their hosts is critical to both colonization and invasive disease. Infection with *Neisseria gonorrhoeae* (the gonococcus, Gc) is characterized by the influx of neutrophils [polymorphonuclear leukocytes (PMNs)], which fail to clear the bacteria and make antimicrobial products that can exacerbate tissue damage. The inability of the human host to clear Gc infection is particularly concerning in light of the emergence of strains that are resistant to all clinically recommended antibiotics. Bacterial metabolism represents a promising target for the development of new therapeutics against Gc. Here, we generated a curated genome-scale metabolic network reconstruction (GENRE) of Gc strain FA1090. This GENRE links genetic information to metabolic phenotypes and predicts Gc biomass synthesis and energy consumption. We validated this model with published data and in new results reported here. Contextualization of this model using the transcriptional profile of Gc exposed to PMNs revealed substantial rearrangements of Gc central metabolism and induction of Gc nutrient acquisition strategies for alternate carbon source use. These features enhanced the growth of Gc in the presence of neutrophils. From these results, we conclude that the metabolic interplay between Gc and PMNs helps define infection outcomes. The use of transcriptional profiling and metabolic modeling to reveal new mechanisms by which Gc persists in the presence of PMNs uncovers unique aspects of metabolism in this fastidious bacterium, which could be targeted to block infection and thereby reduce the burden of gonorrhea in the human population.

**IMPORTANCE:**

The World Health Organization designated Gc as a high-priority pathogen for research and development of new antimicrobials. Bacterial metabolism is a promising target for new antimicrobials, as metabolic enzymes are widely conserved among bacterial strains and are critical for nutrient acquisition and survival within the human host. Here we used genome-scale metabolic modeling to characterize the core metabolic pathways of this fastidious bacterium and to uncover the pathways used by Gc during culture with primary human immune cells. These analyses revealed that Gc relies on different metabolic pathways during co-culture with human neutrophils than in rich media. Conditionally essential genes emerging from these analyses were validated experimentally. These results show that metabolic adaptation in the context of innate immunity is important to Gc pathogenesis. Identifying the metabolic pathways used by Gc during infection can highlight new therapeutic targets for drug-resistant gonorrhea.

## INTRODUCTION

*Neisseria gonorrhoeae* (the gonococcus, Gc) is the causative agent of the sexually transmitted infection gonorrhea. Gc is a human specific pathogen that is uniquely adapted to colonize human mucosal surfaces, where it survives despite initiating a robust inflammatory response and influx of innate immune cells, specifically polymorphonuclear leukocytes (PMNs or neutrophils) (1). The mechanisms that Gc uses to resist PMN clearance remain incompletely understood. Gc encodes a relatively small repertoire of virulence factors compared to other pathogenic bacteria, and it has no known exotoxins (2). Instead, the success of Gc during human infection is related to its physiology, in particular its ability to exploit the resources in the host environment. Gc is a metabolic specialist that exhibits a limited carbon source preference, growing only on glucose, lactate, and pyruvate as sole carbon sources, suggesting that these nutrients are provided by the human host (3). As a human-adapted pathogen, many of the molecular determinants driving the specificity of the human host are required for nutrient acquisition. Metabolic gene products involved in lactate acquisition, nutrient metal import, and anaerobiosis are all required for full Gc virulence in models of infection ranging from cell culture to murine genital colonization to experimental human urethral challenge (4 - 11). However, many aspects of Gc metabolism remain undefined, such as the nutrients used by Gc in different infectious contexts and the core metabolic pathways required to sustain infection.

*Ge*nome-scale metabolic *n*etwork *re*constructions (GENREs) are a mathematical framework encompassing much of the known metabolic information on an organism. A draft GENRE can be generated with an annotated genome and several automated network reconstruction tools (12 - 15), then extensively manually curated using published literature and experimental data. GENREs can simulate all possible growth capabilities of an organism, which are then constrained by biological and physical parameters such as metabolite availability and optimized for a desired outcome, such as biomass production. GENREs enable large-scale, *in silico* manipulations of bacterial metabolism and have been used in a variety of applications including genome-wide knockout screens, synthetic lethal studies, and metabolic engineering that would otherwise be time-consuming and labor intensive to conduct (16). More recently, these tools have been used for the integration and interpretation of multi-omics data and applied to studies of human health and disease, including modeling of the metabolism of prominent human pathogens including *Mycobacterium tuberculosis*, *Staphylococcus aureus*, *Pseudomonas aeruginosa*, *Clostridioides difficile*, and *Salmonella typhimurium* (17 - 22). In contrast, there is no published model of Gc metabolism; while there is a GENRE for the related *Neisseria meningitidis* (23), these two species are known to have key differences in their metabolism, for instance in sugar utilization (24). Moreover, few studies to date have applied metabolic modeling to pathogens in the context of immune cells, leaving a gap in knowledge of how immune-driven metabolic shifts shape bacterial metabolism. Systems-biology approaches are well suited to interrogating complex metabolic network interactions between organisms (25). These factors together make metabolic modeling an ideal platform for understanding novel metabolic drivers of Gc virulence.

Here, we present iNgo_557, a GENRE of Gc metabolism. This model enables the prediction of carbon source utilization and growth yields that recapitulate the behavior of Gc when grown in rich media. Metabolic network coverage in iNgo_557 includes genes, reactions, and metabolites that were initially identified by homology to a model of *N. meningitidis* and further curated using an automated model with support from literature evidence. The quality of iNgo_557 was further enhanced by an update of standardized formatting and improving annotations. iNgo_557 was validated by comparing phenotypic predictions to experimental data sets and benchmarked with the MEMOTE test suite for assessing reconstruction quality. iNgo_557 was then contextualized with transcriptomic data that we generated for Gc grown with and without exposure to PMNs [Gene Expression Omnibus (GEO) database GSE123434], from which we identified and characterized unique metabolic features of the bacteria during an innate immune challenge. This GENRE of a clinically important, metabolically fastidious bacterium is a new resource for the *Neisseria* and microbial metabolic modeling communities. The insights into immune-driven metabolic shifts in Gc revealed by this transcriptionally guided GENRE can inform the future development of therapeutic strategies to combat antibiotic-resistant gonorrhea.

## RESULTS

### A genome-scale network reconstruction of *Neisseria gonorrhoeae* metabolism

**Fig 1:**
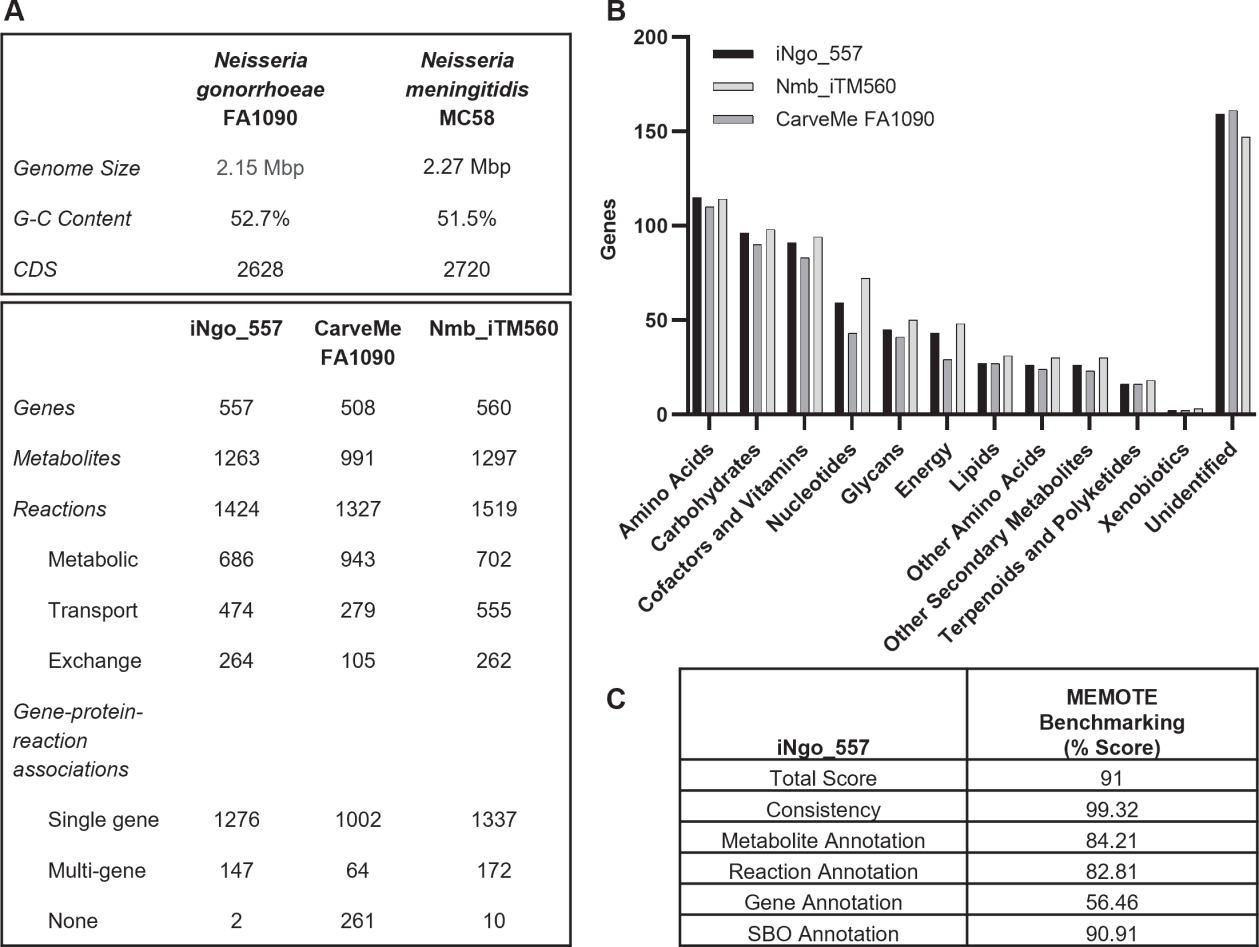
Genome-scale metabolic model of Gc strain FA1090. (**A**) (Upper panel) Comparison of Gc strain FA1090 and *N. meningitidis* strain MC58. (Lower panel) Properties of iNgo_557, CarveMe FA1090, and Nmb_iTM560. (**B**) Comparison of KEGG functional annotations for genes present in the three models. Some genes have multiple functions and are assigned to multiple categories. (**C**) MEMOTE benchmarking scores of iNgo_557.

We generated iNgo_557, a genome-scale metabolic network reconstruction of Gc strain FA1090, the type strain of Gc which is widely used and highly annotated. A published reconstruction of *N. meningitidis* M58 (Nmb_iTM560) served as the starting point (23) (Fig. 1A). Nmb_iTM560 was based on the highly annotated iAF1260 reconstruction for *Escherichia coli* and was built using the Biochemical, Genetic and Genomic (BiGG) knowledge base framework (26). We identified homologous genes between *N. meningitidis* M58 (AE002098.2) and *N. gonorrhoeae* FA1090 (AE004969.1) using a homology matrix–based workflow for generating high-quality multi-strain genome-scale metabolic models (27). Gc and *N. meningitidis* were found to share significant homology across large stretches of the genome, particularly for metabolic genes: of the 560 genes, 1,519 reactions, and 1,297 metabolites originally present in Nmb_iTM560, 494 genes, 1,223 reactions, and 1,189 metabolites were preserved in iNgo_557 based on homology (Data Set S1). Orphan reactions from Nmb_iTM560 with no corresponding gene were included in the initial Gc reconstruction and de-orphaned or removed where possible during manual curation. The format was updated to SBML Level 3, the most up-to-date community standard (28). Gene, reaction, and metabolite annotations were updated from KEGG, PATRIC, UniProt, MetaNetX, MetaCyc, PubMLST, and BiGG databases wherever possible (26, 29 - 34).

Characteristics of the original Nmb_iTM560 model were conserved, including the presence of a periplasmic compartment, simplified cytochrome respiration pathways, iron acquisition pathways from ferric iron and host proteins, and a biomass equation that reflects the neisserial cell composition, including DNA, RNA, and protein production, growth-associated ATP, and ATP maintenance requirements. Targets for manual curation of the automated reconstruction in iNgo_557 included complete resolution of mass and charge balance inconsistencies, the resolution of import and export loops, removal of carbohydrate import through the phosphotransferase system (which is not functional in pathogenic *Neisseria*), addition of amino acid catabolism pathways, curation of lipooligosaccharide synthesis for Gc and its addition to the biomass equation, modification of the biomass composition for Gc where appropriate, and simplification of lipid biosynthesis (Data Set S1 and S2). Additionally, in Nmb_iTM560, catalytic cofactors such as biotin, thiamine pyrophosphate, pyridoxal-5-phosphate, iron, zinc, manganese, NAD, and flavin adenine dinucleotide were included as consumed reactants in reactions to reflect biological requirements for biomass production (35). While useful, the presence of these artificially consumed cofactors causes all cofactor-containing reactions in the model to be imbalanced and impedes the accurate assessment of reaction stoichiometries in the reconstruction using automated modeling tools. Furthermore, a recent reassessment of biomass equations in GENREs found that the inclusion of universally essential cofactors in the biomass equation improved gene essentiality predictions (36). These catalytic cofactors were removed in iNgo_557 as reactants from each reaction and added instead to the biomass equation.

This homology-based reconstruction process was incapable of identifying Gc-specific genes that were not present in Nmb_iTM560 (27). Therefore, to expand the metabolic coverage of iNgo_557 for metabolic pathways that are unique to Gc, genes and their corresponding reactions/metabolites were added from an automated reconstruction in the BiGG namespace of Gc FA1090 that was generated using CarveMe (37). Each of the unique genes identified by CarveMe was manually evaluated. Of the 508 genes with metabolic functions predicted by CarveMe, 388 were already present in the model. CarveMe identified an additional 39 genes and corresponding reactions that were supported by manual evaluation of the literature, and they were subsequently included in iNgo_557 (Fig. 1A; Data Set S1) (37). The remaining 81 genes identified by CarveMe did not have sufficient evidence to support the assigned metabolic function and were not added (Data Set S1).

iNgo_557 is available as an Excel file in Data Set S3 and on Github, including as an SBML file. A comparison of the overall functions captured by iNgo_557 compared to Nmb_iTM560 and CarveMe automated models, as assessed by KEGG reaction categories, is presented in Fig. 1B. The overall quality of the reconstruction was assessed using MEMOTE (38). MEMOTE scores serve as a metric for benchmarking consistency in reaction stoichiometries, annotations, and adherence to community standards. Each category in MEMOTE received a score between 82% and 99% except for gene annotation (56.46%), which reflects the limited information in current gene annotation repositories for Gc. The cumulative MEMOTE score of iNgo_557 was 91%, representing an improvement over the CarveMe automated model (23%) (Fig. 1C).

### Validation and utility of predictions in iNgo_557 with experimental phenotypes

**Fig 2:**
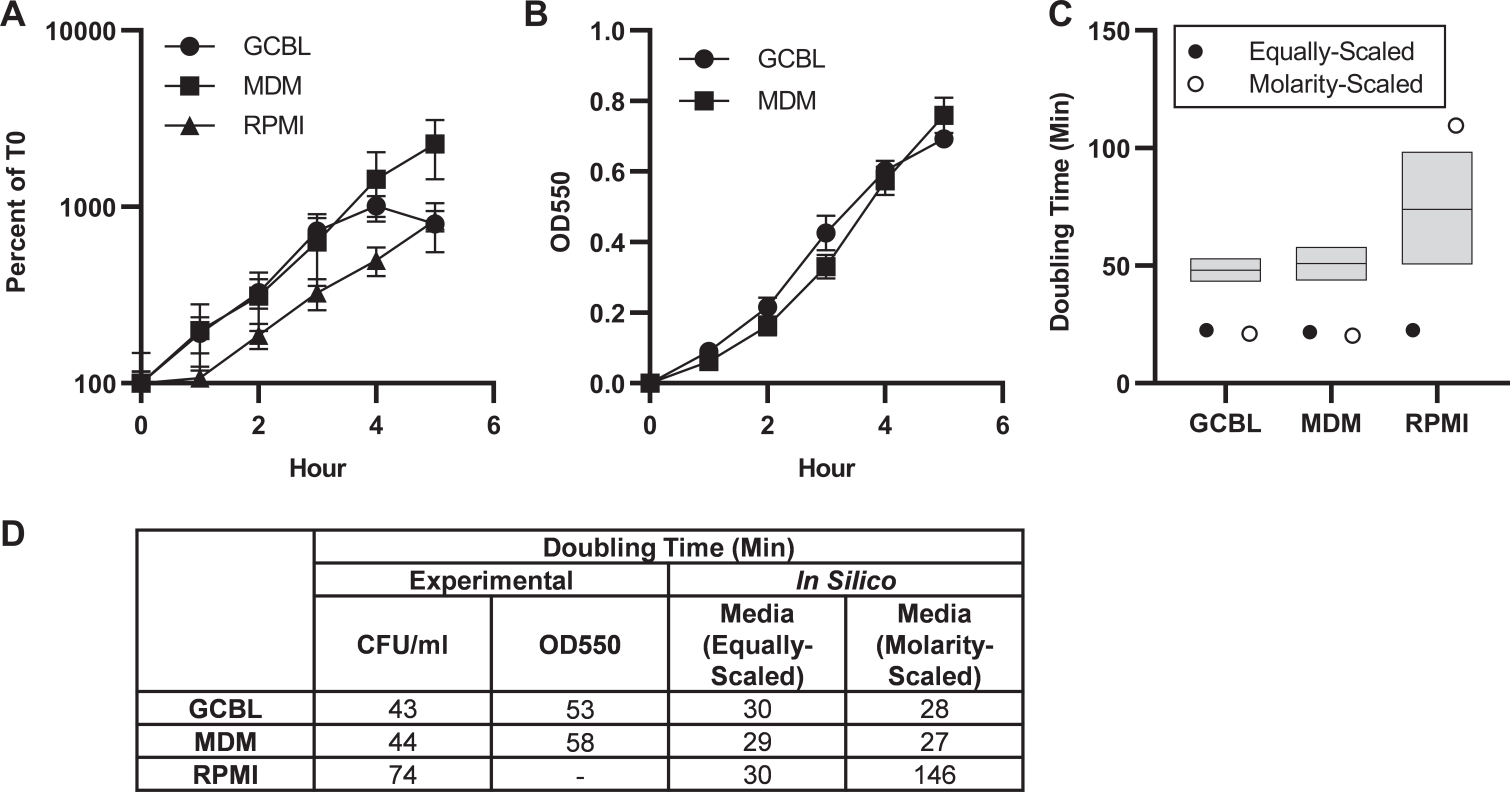
iNgo_557 predicts doubling times that reflect relative growth of Gc in three culture media. Log phase WT Gc was backdiluted into GCBL, MDM, or RPMI and grown over 5 hours. Growth was monitored by (**A**) enumeration of CFU/mL, reported as percent of CFU measured at 0 hour or (**B**) optical density at 550 nm. Optical density for Gc grown in RPMI was not determined due to the presence of phenol red indicator that interfered with the readings. *N* = 4–5 biological replicates. Symbols represent the mean. Error bars represent SEM. (**C and D**) Doubling time from A and B was calculated for Gc grown in each medium using GrowthCurver. (**C**) Experimentally determined doubling times for CFU/mL were compared for each media to the predicted doubling times *in silico* using the equivalent *in silico* media. The boxplot represents the maximum, median, and minimum experimentally observed doubling times. (**D**) Numerical values for experimentally determined (mean) and *in silico* predicted doubling times.

*In silico* predictions of biomass flux and amino acid supplementation for iNgo_557 were performed and compared to experimental data to validate the model. First, the compositions of three media used for Gc growth were determined: Gonococcal Base Liquid (GCBL), Morse’s Defined Media (MDM), and Roswell Park Memorial Institute 1640 media (RPMI). The metabolites present in each media were assigned to corresponding model exchanges in equivalent amounts and deemed “equally scaled” media (Data Set S4). These simulated media were used to compute biomass flux and consequent predictions of Gc doubling time. Doubling time predictions made with iNgo_557 were then compared to experimental values by conducting growth curves of FA1090 Gc in each of these media (Fig. 2A and B). The bacterial doubling times predicted by iNgo_557 for equally scaled media were within 13, 15, and 34 minutes of experimentally determined values in GCBL, MDM, and RPMI, respectively (Fig. 2C and D). All predicted doubling times were faster than what was measured experimentally, which is consistent with the structuring of metabolic network models to predict optimal growth (39).

As shown in Fig. 2A, growth on RPMI was the slowest experimentally, reflecting the limited nutrient content in this media relative to MDM and GCBL (Data Set S4). Specifically, metabolite concentrations in RPMI are ~2- to 10-fold less than the concentrations in MDM and GCBL. For example, glucose is found in MDM and GCBL at 27.8 and 22.2 mM, respectively, but in RPMI at 11.1 mM (Data Set S4). Based on these differences, these three media were “molarity scaled” for simulation in iNgo_557, in which exchanges were set to be equal to the molarity of each respective metabolite in the media to better represent relative availability, as has been done previously (40) (Fig. 2C and D). While using molarity-scaled media for the substrate uptake constraints did not change growth predictions for Gc in MDM and in GCBL, the predicted doubling time of Gc in RPMI was substantially slowed, from 30 to 146 minutes (Fig. 2C and D).

**Fig 3:**
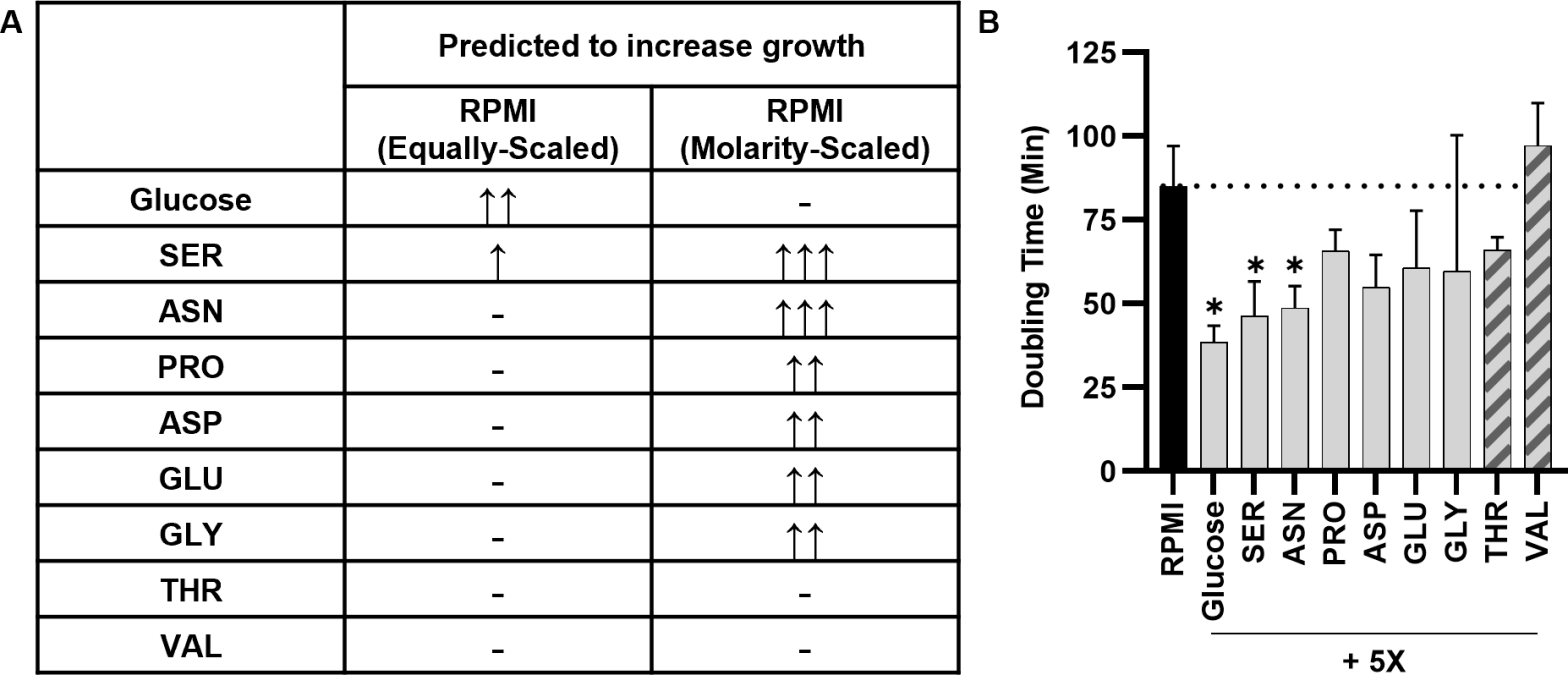
Identification of nutrients that limit Gc growth in RPMI. (**A**) Metabolites in RPMI that are predicted using iNgo_557 to increase Gc growth when increased by 5× the standard flux. Increase in doubling time represented by ↑ ≥ 10%, ↑↑ ≥ 20%, and ↑↑↑ ≥ 30%. (**B**) WT Gc was grown in RPMI supplemented with 5× the concentration of the indicated metabolites for 5 hours. Growth was monitored by enumeration of CFU/mL, and doubling time was calculated using GrowthCurver. Results are from *n* = 3 biological replicates. Bars represent the mean. Error bars represent SEM. Dotted line indicates doubling time in unmodified RPMI (black bar). Metabolites predicted to increase growth are in gray bars; control metabolites predicted to not increase growth are in hatched bars. *P < 0.05 by one-tailed *t*-test is relative to unmodified RPMI.

To identify the substrate(s) that were limiting for Gc growth in RPMI compared with MDM or GCBL, we used iNgo_557 to predict the doubling time of Gc in a revised formulation of RPMI where the constraint for uptake of each component was individually increased to fivefold the original medium (Table S1). In the simulation of growth in equally scaled RPMI, only glucose and serine were individually predicted to increase Gc growth when supplemented at 5× the original constraint (Fig. 3A; Table S2). In the simulation of molarity-scaled RPMI, serine, asparagine, proline, aspartate, glutamate, and glycine were individually predicted to increase Gc growth when supplemented at 5× the original constraint (Fig. 3A; Table S2). We tested these predictions experimentally. Addition of 5× glucose, serine, or asparagine to RPMI, which was predicted to increase Gc growth the most, significantly increased the growth rate of Gc compared with unmodified RPMI (Fig. 3B). Addition of proline, aspartate, glutamate, or glycine, which had the smallest predicted increase in growth, did not significantly enhance growth compared to unmodified RPMI. The experimental growth rate of Gc was not significantly affected when RPMI was supplemented with 5× threonine or valine, which were not predicted to increase growth (Fig. 3B). These findings demonstrate that iNgo_557 can help predict potential nutrients that stimulate Gc growth.

Gc is reported to be capable of growing on only three carbon sources: glucose, lactate, and pyruvate (3). While iNgo_557 successfully predicted the growth of Gc on glucose, lactate, and pyruvate as carbon sources in molarity-scaled MDM (27, 43, 28 minutes of doubling times), it also predicted slow growth of Gc in their absence (247 minutes of doubling time). This slow growth is due to the predicted catabolism of amino acids by Gc, for which Gc encodes the required enzymes. However, there was no significant growth of Gc in MDM that did not have glucose, lactate, or pyruvate added (Fig. S3A), even with additional amino acid supplementation (Fig. S3B). Metabolomics and C^13^ metabolic flux analysis recently conducted on Gc strain MS11 demonstrated that Gc consumes a variety of amino acids when grown in a glucose-containing medium that is a derivative of MDM (41). To further explore Gc usage of amino acids, we compared the simulated growth of Gc in molarity-scaled MDM (Fig. S4) to the C^13^ distributions reported in reference (41). Our *in silico* predictions of metabolic flux were consistent with the reported C^13^ distributions: Gc exhibited a bipartite metabolism in which carbon from glucose was metabolized to acetyl-CoA, and amino acids were independently metabolized via the tricarboxylic acid cycle (TCA) cycle. Thus, a prediction made by our model, that Gc can catabolize amino acids, is supported by experimental evidence. To account for Gc usage of amino acids in the presence of its known carbon sources, amino acid catabolic pathways were left intact in iNgo_557.

We compared gene essentiality predictions yielded by iNgo_557 on GCB with a published data set comprising essential genes, which were identified through the growth of strain MS11 transposon insertion mutants (transposon sequencing, TnSeq) on GC agar (Data Set S5) (42). In iNgo_557, a gene was predicted to be essential if less than 10% of the optimal biomass of the wild type (WT) could be produced by a mutant in single-gene deletion simulations. Predicted gene essentiality had an agreement of 73% with a Mathews correlation coefficient (MCC) of 0.43 (Data Set S5). Genes identified as essential by both iNgo_557 and TnSeq included those related to lipooligosaccharide and peptidoglycan biosynthesis, purine metabolism, and pyruvate metabolism. Of the genes identified as non-essential by iNgo_557 but essential by TnSeq, many encoded participants in pyrimidine metabolism, oxidative phosphorylation, and glycolysis. One such gene, encoding pyruvate kinase (*pyk*), was predicted by iNgo_557 as non-essential, but essential by TnSeq. We verified that *pyk* could be deleted from Gc and that the resulting null mutant could grow in GCBL containing glucose as the sole carbon source, albeit slower than the WT parent or when pyruvate was provided (Fig. S5). While Tnseq has been used for model validation in the past, a major caveat of using it is that genes determined to be essential by TnSeq may only appear so in a competitive setting when mixed with a library of other transposon mutants (43, 44). Differences in media composition and bacterial strain backgrounds can also contribute. For these reasons, we did not use TnSeq gene essentiality data for further curation of iNgo_557.

Taken together, there was good concordance between Gc growth predicted using iNgo_557 and experimental results generated in this study and from previously published work. We conclude that iNgo_557 can be used to predict growth dynamics of Gc in various *in vitro* conditions, which can be further validated experimentally.

### Transcriptome-guided modeling of Gc metabolism during co-culture with primary human neutrophils predicts a shift in the pyruvate axis

GENREs serve as a tool for scaffolding complex metabolic information in human-interpretable formats. One such application is the integration of transcriptional data with GENREs to develop a comprehensive picture of bacterial metabolism in complex and uncharacterized environments (45). Given that Gc is a human-specific pathogen, we sought to use the reconstruction to predict metabolic phenotypes that are consistent with Gc growth in the context of human neutrophils (PMNs), the predominant immune cell that is recruited during infection. To investigate how Gc metabolism shifts in response to co-culture with PMNs, transcriptomic data from Gc co-incubated with PMNs for 1 hour were integrated with iNgo_557 to generate contextualized models that offer insight into the metabolic state of Gc during infection.

To accomplish this goal, we applied the RIPTiDe (Reaction Inclusion by Parsimony and Transcript Distribution) algorithm, which uses RNA sequencing (RNA-seq) data to identify the most cost-effective usage of metabolism while also reflecting the organism’s transcriptional investment. In brief, the model is iteratively constrained to a fraction of the maximal possible biomass. The algorithm then assigns linear coefficients to weight model reactions based on transcript abundance. Reactions corresponding to highly abundant transcripts are favored to be retained, and low abundance transcripts are favored to be removed (i.e., “pruned”) from the model. Consistency of the model with the transcriptome is assessed by Spearman correlation coefficient. The fraction with the highest correlation is selected as the context-specific model (Fig. S6). RIPTiDe has been used successfully with models of *P. aeruginosa* and *C. difficile* to uncover metabolic contributors to virulence in the context of mucin degradation, biofilm formation, murine infection models, and co-culture with other microbes (17, 19, 46). We reasoned this approach would generate context-specific models of the metabolism of Gc when grown with and without PMN co-culture and would identify those reactions that are likely to be differentially active in each condition.

The transcriptome data set we used was from RNA-seq of a constitutively opacity protein (Opa)–deficient isolate of strain FA1090 Gc, which was cultured in RPMI + 10% fetal bovine serum (FBS) for 1 hour. Gc was cultured in the presence or absence of primary human PMNs that were adherent and treated with the chemokine interleukin-8 to reflect the activated state of immune cells during infection (47). This Opa-negative isolate of Gc survives significantly better when exposed to PMNs than Opa-expressing strains that are rapidly internalized and killed by PMNs (48). RIPTiDe generated two context-specific models of Gc metabolism: one for Gc in a medium without PMNs and one for Gc with PMNs (Fig. S6). For each of the two models, flux samples were generated to assess all possible metabolic profiles in the two environmental contexts. Flux samples generated with the models significantly correlated with the transcript abundances derived from RNA-seq for each condition (*r* = 0.242, *P* < 0.001 for Gc without PMNs, and *r* = 0.263, *P* < 0.001 for Gc with PMNs) (Fig. S6). These *r* values are consistent with those obtained from models of other organisms to which RIPTiDe was applied (17), indicating that the context-specific metabolic profiles predicted with RIPTiDe align with experimental data.

**Fig 4:**
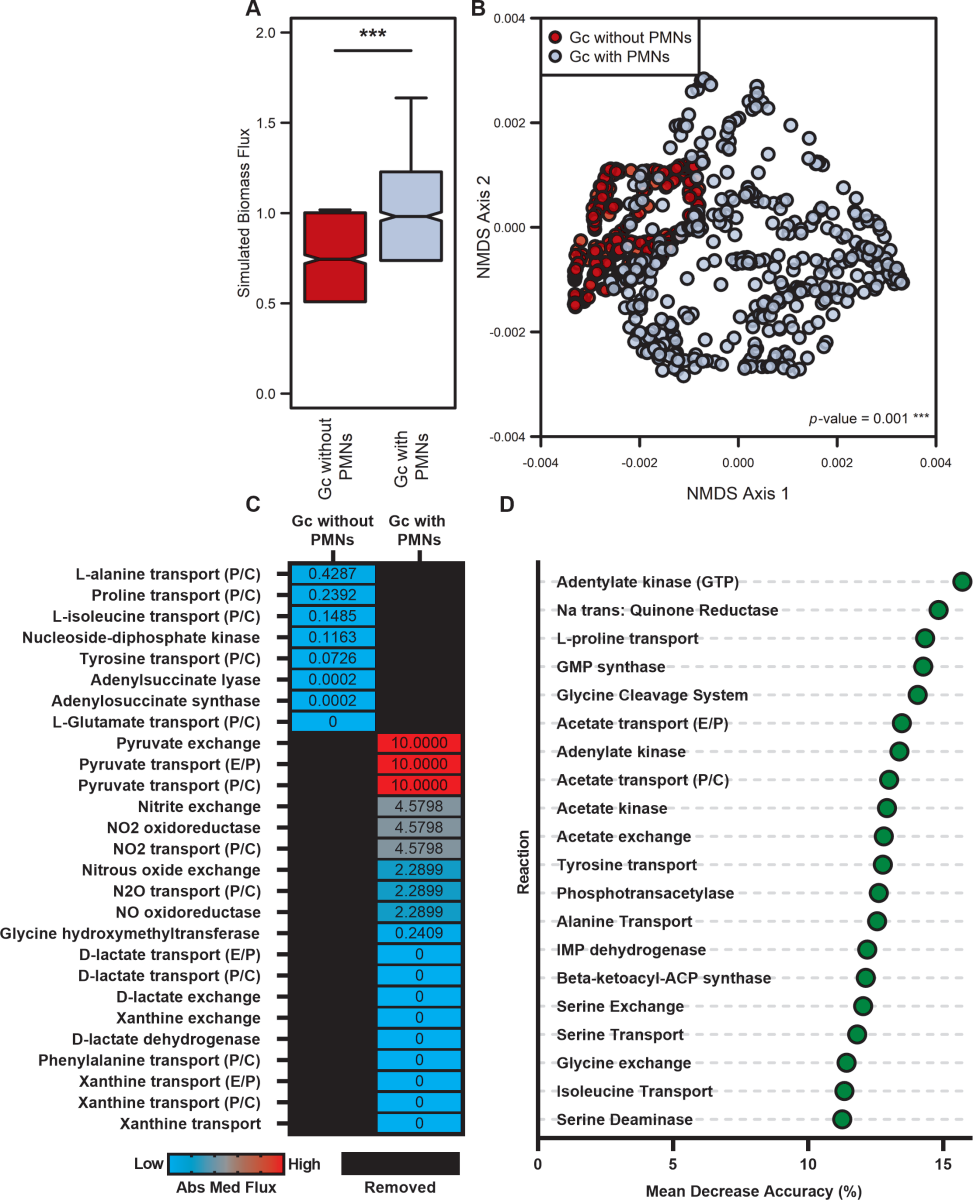
Metabolic activity predictions differ between Gc cultured without PMNs and Gc co-cultured with PMNs. Transcriptomes from Gc cultured with and without PMNs for 1 hour were used to generate context-specific models of iNgo_557 using RIPTiDe. Inactive reactions were pruned during contextualization. (**A**) Boxplot of biomass objective flux distributions (*n* = 500) from each context-specific model. Significance determined by Wilcoxon rank-sum test (*P* value < 0.001). (**B**) Axes 1 and 2 of four-dimensional Bray-Curtis-based NMDS ordination for flux sampling results from non-biomass reactions shared between context-specific models of iNgo_557. Significant difference determined by PERMANOVA. (**C**) The median absolute value of reaction activities for uniquely active metabolic reactions in each context-specific model (*n* = 1,000). Black boxes indicate reactions are absent in the corresponding model and carry no flux. Median absolute values of 0 indicate that the reaction carried flux, but the median of flux from 1,000 simulations for that reaction was 0. (**D**) Random Forest supervised machine learning (49, 50) was used to categorize flux sample activity as Gc without PMNs and Gc with PMNs for non-biomass metabolic reactions shared between the contextualized models. The mean decrease accuracy, which predicts the impact of removal of the reaction from the model on Random Forest categorization predictions (Gc without PMNs vs Gc with PMNs), for the top 20 most differentiating reactions is shown.

Biomass flux was significantly increased in the contextualized model of Gc co-cultured with PMNs, compared with Gc cultured without PMNs (Fig. 4A), suggesting an overall stimulation of Gc metabolism in the presence of PMNs. Flux distributions for each model were then compared using non-metric multidimensional scaling (NMDS) of consensus reactions shared between both models to broadly identify metabolic growth patterns used by the context-specific models. NMDS revealed that the sampled flux distribution for Gc co-cultured with PMNs overlapped with, but was distinct from, the sampled flux distribution for Gc cultured without PMNs (Fig. 4B). This result reflects that the media used for growth is consistent between the two models, but co-culture with PMNs caused a shift in metabolic pathways used for growth.

We further analyzed the contextualized models to better understand the shifts in metabolism that resulted in the distinctions observed in the NMDS analysis. Reactions unique to each model (non-consensus reactions) were identified, and the absolute median activity for each reaction was determined for 1,000 simulations to examine the contribution of each reaction to biomass production (Fig. 4C). From this analysis, we identified a set of 19 reactions that were unique to Gc co-cultured with PMNs and eight reactions unique to Gc cultured without PMNs. Several reactions involved in metabolite import and catabolism were unique to Gc co-cultured with PMNs, suggesting that there are changes to the metabolites available to Gc in this condition, possibly due to the competition with or excretion by PMNs. Specifically, pyruvate and d-lactate exchange reactions were unique to Gc co-cultured with PMNs (Fig. 4C). Further, the model predicts that Gc imports these metabolites (Fig. S7), suggesting bacterial use of these alternative carbon sources in the presence of PMNs. This observation aligns with extensive evidence that PMNs secrete lactate as a byproduct of oxidative metabolism, which stimulates Gc growth (4, 51). Similarly, Gc co-cultured with PMNs was also predicted to uniquely carry flux through nitrogen metabolism, in particular the import of nitrite, nitrite oxidoreductase, nitric oxide reductase, and nitrous oxide export (Fig. 4C; Fig. S7). These findings align with the reported production of nitric oxide (NO) via inducible nitric oxide synthase in stimulated PMNs (52). Although NO is used by phagocytes to directly kill pathogens, Gc can exploit this aspect of inflammation by detoxifying NO to nitrous oxide or using nitrite and nitric oxide as terminal electron acceptors during anerobic growth (53). Together, these observations support the hypothesis that neutrophil byproducts mediate remodeling of Gc metabolism.

**Fig 5:**
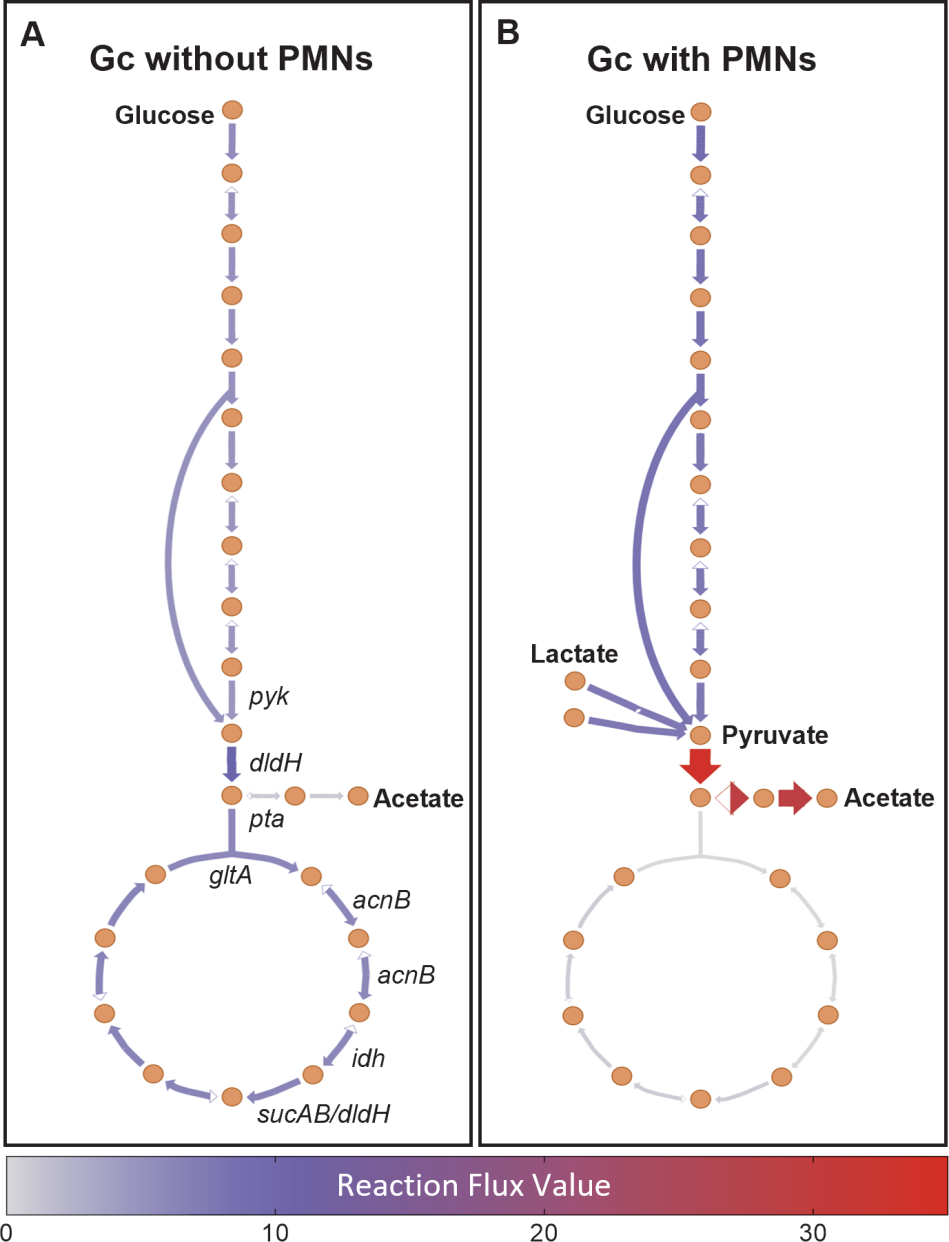
Visualization of flux balance analysis for central carbon metabolism in contextualized models of Gc with and without PMNs. Orange circles indicate metabolites. Relevant imported and exported metabolites are indicated in bold. Arrows indicate reactions. The intensity of coloration and the arrow size indicate the degree of flux through reactions. Conditionally essential genes corresponding to reactions are indicated in italics. Schematics were generated with Escher.

We next assessed reactions that were shared between both models of Gc cultured without PMNs and Gc co-cultured with PMNs but carried different levels of flux. From this, we identified reactions that most strongly discriminated between the metabolic activity of the two models. This analysis employed a supervised machine learning approach with Random Forest, a categorization algorithm that can segregate flux samples based on the contextualized models (Fig. 4D) (50). In brief, we trained a Random Forest classifier to predict reactions that were critical in defining whether the contextualized model was associated with culturing with or without PMNs. Specifically, the Random Forest input was reaction flux distributions for consensus reactions between the contextualized models from the flux samples (*n* = 500). The classifier had an out-of-bag accuracy above 98%, indicating that membership for each contextualized model can robustly be predicted from reaction content within the two contextualized models. We then assessed mean decrease accuracy (MDA) to identify reactions that, when removed from the model, most affected the categorization predictions of the Random Forest. Gc grown in the presence and absence of PMNs was particularly distinguished by flux out of the pyruvate node, through acetate synthesis. Specifically, acetate exchange, acetate transport, acetate kinase, and acetate phosphotransacetylase were identified as reactions that impacted the categorization capabilities of the Random Forest (MDA ~13%) (Fig. 4D). Acetate production is a prominent feature of bacterial overflow metabolism, in which ATP is generated from the production of acetate from acetyl-CoA via the phosphate acetyl-transferase and acetate kinase (PTA-AckA) pathway rather than shuttled into carbon backbones for biomass (54). Visualization of flux balance analysis (Fig. 5A and B) demonstrated a predicted increase in acetate flux in co-culture with PMNs, consistent with increased carbon flux from the addition of alternative carbon sources, such as lactate and pyruvate.

Conditionally essential genes were predicted by conducting essential gene calculations in each model, then comparing them (Table 1; Data Set S7). Twelve genes were predicted to be essential only when Gc was cultured without PMNs, and two genes were predicted to be essential only when Gc was co-cultured with PMNs. Of the 12 genes predicted to be essential only when Gc was cultured without PMNs, seven are within a single pathway exiting the pyruvate synthesis node (Table 1): pyruvate kinase (*pyk*), portions of the pyruvate dehydrogenase complex (*dldH*), phosphate acetyltransferase (*pta*), citrate synthase (*gltA*), aconitase (*acnB*), and isocitrate dehydrogenase (*idh*) were all predicted to be essential only for Gc cultured without PMNs.

**TABLE 1:** Conditionally essential genes predicted by single-gene deletion analysis of contextualized models of Gc without PMNs and Gc with PMNs

Ngo ID	Annotation	Gene ID
Gc co-cultured with PMNs		
NGO0799	Inosine-5-monophosphate dehydrogenase	*imdH*
NGO2164	GMP synthase	*guaA*
Gc cultured without PMNs		
NGO0214	Phosphate acetyltransferase	*pta*
NGO0562	Dihydrolipoamide dehydrogenase	*dldH*
NGO0918	Citrate synthase	*gltA*
NGO0925	Dihydrolipoamide dehydrogenase	*dldH*
NGO1082	Isocitrate dehydrogenase	*Idh*
NGO1231	Aconitate hydratase	*acnB*
NGO1325	Glycine dehydrogenase	*gcvP*
NGO1404	Glycine cleavage system protein H	*gcvH*
NGO1406	Glycine cleavage system protein T	*gcvT*
NGO1470	NAD(P) transhydrogenase subunit alpha	*pntA*
NGO1472	NAD(P) transhydrogenase subunit beta	*pntB*
NGO1881	Pyruvate kinase	*pyk*

**Fig 6:**
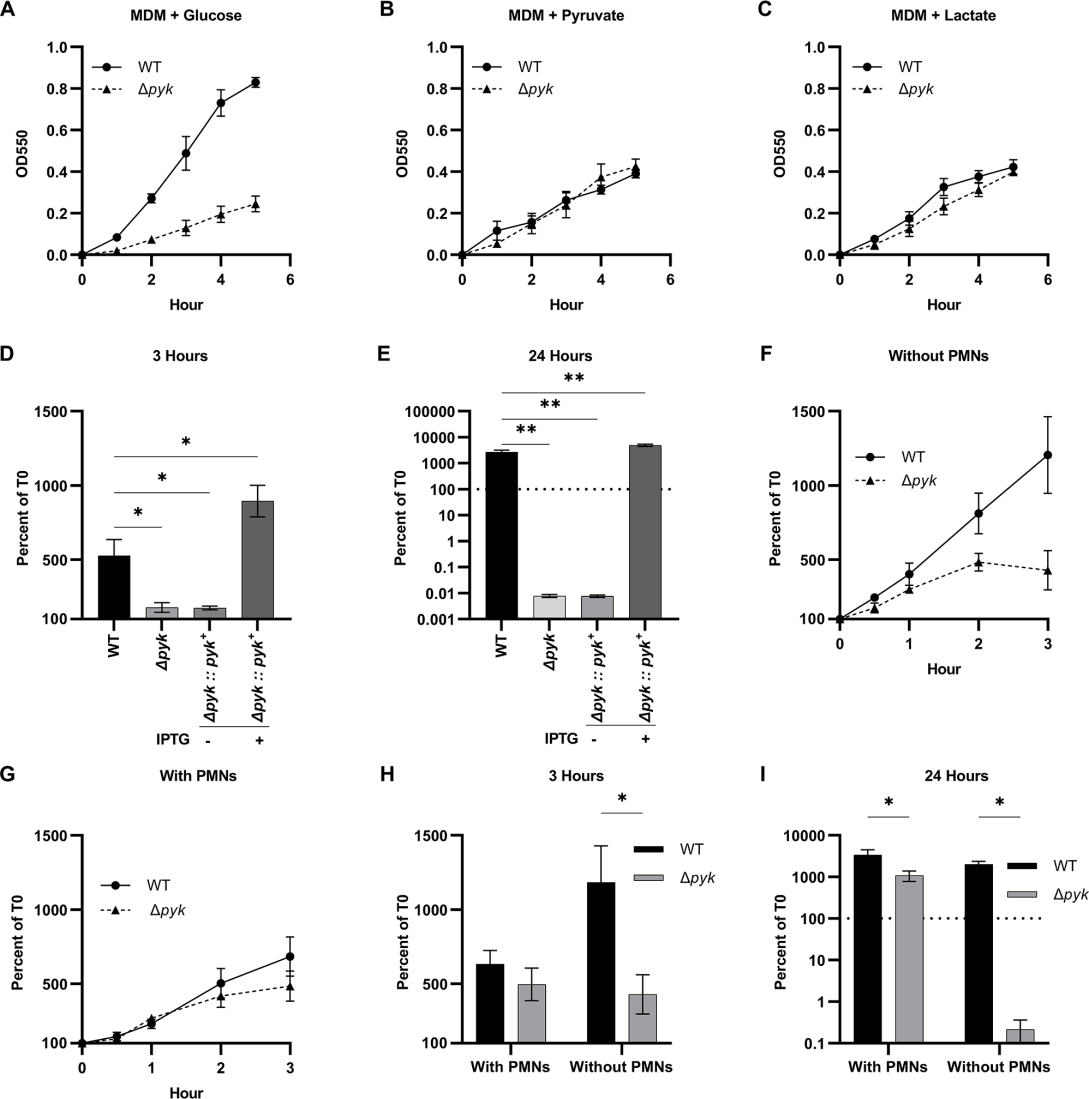
Pyruvate kinase is conditionally essential for *N. gonorrhoeae* in glucose-containing medium but not for bacteria cultured with PMNs. WT Gc and isogenic Δ*pyk* mutant were cultured in MDM containing (**A**) glucose, (**B**) pyruvate, or (**C**) l-lactate as the primary carbon source. Growth over 5 hours was monitored by optical density at 550 nm for *n* = 3 biological replicates. Symbols represent the mean. Error bars represent SEM. (**D**) and (**E**) WT, Δ*pyk*, and Δ*pyk* expressing an IPTG-inducible *pyk* open reading frame were inoculated in RPMI +10% FBS. 1 mM IPTG was added where indicated. CFUs were enumerated at (**D**) 3 and (**E**) 24 hours, and Gc growth is reported relative to CFU for that strain at 0 hour (100%). Bars represent the mean. Error bars represent SEM. *n* = 3 biological replicates. Significance was determined by one-way ANOVA with Holm–Sidak correction for multiple comparisons, *P < 0.05, **P < 0.01. (**F through I**) WT and Δ*pyk* Gc were exposed to primary human PMNs in suspension or inoculated in RPMI + 10% FBS. CFUs were enumerated at 0.5, 1, 2, 3, and 24 hours, and Gc growth is reported relative to CFU for that strain at 0 hour (100%). (**F and G**) Growth curves without (**F**) and with (**G**) PMNs over 3 hours. (**H and I**) Gc CFU at (**H**) 3 and (**I**) 24 hours, reported as the percent of CFU for that strain at 0 hour (100%). Bars represent the mean. Error bars represent SEM. *n* = 3–4 biological replicates. Significance was determined by two-way ANOVA with Holm–Sidak correction for multiple comparisons, *P < 0.05.

To test the prediction that pyruvate synthesis genes were essential for Gc in rich growth medium but dispensable for Gc in the presence of PMNs, we generated a null mutant in pyruvate kinase (Δ*pyk*), the first enzyme in this pathway. As expected, Δ*pyk* had a growth defect in MDM, GCBL, and RPMI containing glucose as the sole carbon source, while the WT parent grew in these media (Fig. 6A; Fig. S5A and S8). Also as expected, Δ*pyk* and WT Gc grew equally well in MDM containing either lactate or pyruvate as the sole carbon source or when Δ*pyk* was genetically complemented (Fig. 6B through E). We then measured the growth of WT and Δ*pyk* Gc in the conditions used to collect the PMN transcriptomics data. In RPMI + 10% FBS, the Δ*pyk* mutant stopped growing after 3 hours, and by 24 hours its viability had declined to <1% of the inoculum (Fig. 6F, H, and I). In contrast, when cultured in the presence of PMNs, Δ*pyk* Gc grew significantly better than Gc in the absence of PMNs, and in fact increased in viability over 24 hours (Fig. 6G through I). WT Gc grew over this time whether or not PMNs were present (Fig. 6F through I). These results suggest that Gc co-cultured with PMNs has a decreased need for flux through glycolysis and instead imply that Gc has access to alternative carbon sources such as lactate and pyruvate, which support its growth in the presence of PMNs independently of the glycolytic pathway.

## DISCUSSION

Over the last 20 years, genome-scale metabolic modeling has become a powerful tool for context-specific interrogation of complex biological networks. In this study, we developed a highly curated genome-scale metabolic network reconstruction, titled iNgo_557, for Gc strain FA1090. This model predicts the use of glucose, lactate, and pyruvate as carbon sources for Gc, and an increase in growth when selected amino acids are supplemented in a cell culture medium containing one of these carbon sources (3). iNgo_557 was contextualized using transcriptomics data that we recently generated to identify shifts in Gc metabolism that occur in response to co-culture with PMNs. These results represent the first use of genome-scale metabolic modeling in Gc for the discovery of metabolic contributors to virulence.

Through the linkage of gene, reaction, and metabolite information, iNgo_557 facilitates rapid and convenient manipulation of metabolic parameters to identify contributors toward Gc pathogenesis that are otherwise complicated, time-consuming, or laborious to replicate *in vitro*. Independently, GENREs can be used to simulate well-defined environmental contexts, such as growth in laboratory media. iNgo_557 accurately reflects experimental growth phenotypes in simulated media and can be used to predict Gc growth phenotypes following distinct manipulations to these media. We demonstrated one such use: identification of growth-limiting nutrients in RPMI. Other applications include nutrient drop-out experiments, aerobic and anaerobic growth, and gene essentiality studies.

While the predictions generated by our model were consistent with experimental results, incorrect predictions are also informative, revealing points of obscurity in our understanding of Gc metabolism. For example, iNgo_557 predicted the growth of Gc in MDM in the absence of a dedicated carbon source (glucose, lactate, pyruvate). On further interrogation, the predicted growth of Gc on MDM without a carbon source was due to the consumption of serine and alanine as carbon sources. Although Gc encodes the genes necessary to catabolize these amino acids (ALATA_L/ NGO_1047 and SERD_L/NGO_1773 and NGO_0444) and does catabolize these amino acids *in vitro* (41), it is unable to use amino acids as a sole carbon source (Fig. S3). A potential explanation for the inability of Gc to use amino acids as a sole carbon source includes transcriptional regulation, which prevents the use of serine as a sole carbon source in *P. aeruginosa* (55). Post-transcriptional regulation including allosteric modulation of metabolic enzymes (56) and substrate competition for available enzymes or transporters (57) can also prevent the catabolism of amino acids. Our results suggest that a similar form of transcriptional or post-transcriptional regulation may also dictate Gc carbon source utilization. Regulatory features that can impact carbon source usage are missing from GENREs, an inherent limitation in genome-scale metabolic modeling. These discrepancies serve as points for further investigation and facilitate hypothesis generation.

Incorporation of additional layers of regulatory information can improve model accuracy, particularly for the modeling of complex environments such as during co-culture with other species or cell types, which is impeded by the lack of knowledge of the metabolite environment. As a human-adapted mucosal pathogen, Gc must co-exist with a complex assortment of human microbiota, epithelial cells, and mucosal immune cells. The recruitment of PMNs and the inflammation associated with gonococcal infection further complicate an already complex metabolic environment. Transcriptomic integration with metabolic models serves to deconvolute the modeling of these complex settings through unsupervised contextualization of GENREs for a specific environment. As such, we leveraged RIPTiDe with iNgo_557 to better understand the metabolic pathways enabling Gc growth during co-culture with PMNs and to predict the behaviors of this host-associated bacterial species. Intriguingly, several genes are predicted to be essential only when Gc is cultured without PMNs, but not in the context of PMNs, including pyruvate kinase (*pyk*) and pathways exiting the pyruvate synthesis node. Metabolic modeling using iNgo_557 predicts that this effect is due to the bypass of pyruvate synthesis through the import of alternative carbon sources, including lactate and pyruvate, when in the presence of PMNs, which is supported by our growth data. PMNs are highly glycolytic cells that consume glucose and secrete lactate when they are activated (51). The ability of Gc to use lactate would relieve competition between Gc and PMNs for glucose during infection and enable Gc to exploit this byproduct of PMN glycolysis. In support of this possibility, lactate use has been found to be required for Gc survival from PMNs, within cervical epithelial cells, and in the female mouse genital tract (4, 5, 58). The increase in biomass flux predicted for models of Gc cultured with PMNs compared to Gc cultured without PMNs is further consistent with reports that Gc growth on lactate stimulates Gc metabolism (51, 58). Together these results provide evidence that Gc utilizes additional alternative carbon sources, such as lactate and pyruvate, when co-cultured with PMNs to enhance its growth. Future metabolomics studies can measure concentrations of carbon sources and other nutrients at the mucosal sites Gc inhabits to better predict bacterial growth during human infection.

Regardless of the source, carbon exiting the pyruvate synthesis node can proceed in one of the two pathways in Gc: acetate production or oxidation through the TCA cycle. Acetate production through the PTA-AckA pathway is a prominent feature of Gc growth on glucose, lactate, and pyruvate (54, 59). Downstream of pyruvate kinase, iNgo_557 predicted increases in Gc acetate production when in the presence of PMNs. In *N. meningitidis*, acetate is secreted following growth on glucose, lactate, and pyruvate, and the highest activity of the PTA-AckA pathway occurs when all three carbon sources are present, compared with glucose alone (59). Our results are consistent with this observation. Alternatively, glucose, lactate, and pyruvate can instead be further catabolized by the TCA cycle. In *N. meningitidis,* pyruvate dehydrogenase (*dldH*), citrate synthase (*gltA*), aconitase (*acnB*), and isocitrate dehydrogenase (*idh*) reaction activities were all demonstrated to be high in the presence of glucose but decreased in the presence of pyruvate (59). Consistent with the stimulation of these enzymes in the presence of glucose compared to pyruvate, iNgo_557 predicted *dldH*, *gltA*, *acnB*, and *idh* to be essential only in the absence of PMNs, in which glucose is the sole carbon source available. The alleviation of the requirement for *acnB* in the context of PMN co-culture is notable in light of a recent study that identified compensatory mutations within *acnB* that enabled the recovery of antibiotic-resistant *penA* mutant Gc from the mouse genital tract (60). Together our results highlight the pyruvate node as a critical pivot point in Gc metabolism, particularly in the context of an inflammatory environment created by PMNs. Overall, the predictions generated here by contextualized models of iNgo_557 reveal new insights into Gc pathogenesis, highlighting it as a viable platform for the discovery of metabolic pathways associated with virulence and antibiotic resistance.

Treatment options for Gc have become increasingly limited over the last two decades, and only a single recommended antibiotic remains for the treatment of gonorrhea (61). The development of new potential therapies is essential to avoid the threat of completely antibiotic-resistant Gc. Targeting essential bacterial metabolic pathways during infection represents a promising approach, one that was first shown decades ago in the context of sulfonamide antibiotics, which directly inhibit folate synthesis (62). Novel approaches for the treatment of antibiotic-resistant infections have included the application of metabolites to shift the metabolism of pathogens toward a less favorable state (63). There is a need for a revisitation of Gc metabolism and physiology in light of the approaching post-antibiotic era for gonorrhea (64). Technologies such as RNA-seq, forward and reverse genetic screens, and metabolic modeling can all provide insights into Gc metabolism. Here, the integration of transcriptomics with genome-scale metabolic modeling is synergistic, providing more insight into the remodeling of Gc metabolism in the context of PMN co-culture than could be discerned from each technique alone. In sum, this study highlights the opportunities afforded by genome-scale metabolic modeling for the targeted identification of context-specific essential metabolic pathways that enable Gc to thrive within the human host, with further predictions and discoveries remaining to be made.

## MATERIALS AND METHODS

### Genome-scale metabolic reconstruction

To generate a GENRE for Gc, we used *N. meningitidis* M58 Nmb_iTM560 as an initial template for the automated multi-strain model, reconstruction pipeline (27). In brief, the pipeline used bidirectional best hit BLAST to identify genes with >80% homology between *N. meningitidis* M58 (AE002098.2) and *N. gonorrhoeae* FA1090 (AE004969.1) to generate a homology matrix for the two species. A secondary comparison using BLAST on nucleotide sequences was conducted to identify potential homologs with poor open reading frame (ORF) annotation. These automated calls were inspected and reassessed for each gene present in Nmb_iTM560 as indicated in Data Set S1. Using the homology matrix, a draft strain-specific model was generated using COBRApy (65). Metabolic genes (and the corresponding reactions and metabolites) specific to Gc FA1090 were added to the reconstruction using CarveMe when supported by literature evidence (37). Exchange reactions that were missing for extracellular metabolites in the reconstruction were added. The model was then further manually curated to de-orphan reactions and incorporate published metabolic functions for Gc according to literature evidence where possible (Data Set S1). Final gene and reaction calls, along with decision annotations, can be found in Data Set S1. Annotation data were automatically assigned using ModelPolisher (66). Reaction and stoichiometric inconsistencies were corrected for each reaction. All formulas were mass and charge balanced using the BiGG database, when possible, to maintain a consistent namespace (26). A list of mass and charge imbalanced reactions and their corrections are provided in Data Set S1. Additional annotations were collected and added to the annotation field dictionary for all model components from KEGG, PATRIC, UniProt, MetaNetX, MetaCyc, PubMLST, or BiGG databases (26, 29 - 33 - 67). The pipeline for development of the reconstruction is available in the GitHub repository associated with this study (https://github.com/aimeepotter/Gc_GENRE_2022).

### Assessing reconstruction quality

Modeling assessments, including flux balance analysis, flux variability analysis, single-gene knockout analysis, were conducted using COBRApy (65). Model quality was assessed with MEMOTE using a local installation v0.13.0 (38). Gene essentiality predictions were compared to a published data set of essential genes for growth on solid, rich media for Gc strain MS11, which was aligned to Gc FA1090 by bidirectional best-hit BLAST as above. Prediction accuracy was calculated as the number of correct predictions divided by the number of total predictions for genes present in both data sets and the MCC was calculated as in reference (68).

A protocol for defining realistic modeling constraints for *in silico* media was recently described, in which metabolite exchanges are scaled based on the maximum possible usage defined by the concentration of metabolites in millimole per liter (40). We therefore generated two *in silico* exchange reaction constraints for each simulated media: equally scaled, to avoid constraining the model with incorrect assumptions, and molarity-scaled, to match the maximum possible use of metabolites. The concentration of metabolites present in each media and their corresponding assignments to *in silico* media constraints are detailed in Data Set S4. Biomass flux and subsequent doubling times for simulated growth in GCBL, MDM, and RPMI were compared to experimental values. Predictions of Gc doubling time were calculated assuming a biomass equation scaled to 1 g dry weight of bacteria based on the following formula:


$$Doubling\;Time=ln(2{)}^{*}60/(objective\;value)$$

Experimental doubling times were determined using GrowthCurver implemented in R for both optical density (OD) and CFU per milliliter, with stationary phase values trimmed (69).

### RIPTiDe contextualization and analysis

Transcriptomic data retrieved from the GEO database (GSE123434) for Gc cultured without and with PMNs over the course of 1 hour were mapped to the corresponding FA1090 gene IDs using the conversion table provided in Data Set S1 of reference (47). For RIPTiDe contextualization, an unsupervised approach was used in which all exchange reaction bounds were set to ±10, except oxygen, which was set at ±20. The transcriptomic data were then integrated with the model using RIPTiDe using the maxfit_contextualize() function (minimum fraction 0.1, maximum fraction 0.8, *n* = 1,000) to produce contextualized models for Gc grown in the presence or absence of PMNs (70). Flux samples were gathered from consensus reactions between both contextualized models (*n* = 500 samples per model). Bray-Curtis-based NMDS (k = 4, trymax = 25) and permutational multivariate analysis of variance (PERMANOVA) (perm = 999) analyses were accomplished using the Vegan R package (71). Supervised machine learning was accomplished with the implementation of AUC-Random Forest also in R on the sampled flux distributions for shared reactions to determine metabolic functions that distinguish between the two models (ntree = 1,500, importance = TRUE, err.rate = TRUE, mtry = 20), and importance was reported as mean decrease in accuracy for the top 20 predictors (49).

Statistical analysis was performed in R v4.1.0. Visualizations of flux balance analysis were performed using Escher (72).

### Bacterial strains and growth conditions

Opaless Gc is a non-variable Opa-deficient derivative of the FA1090 background constitutively expressing the pilin variant 1-81-S2, which served as the WT for all experiments (48, 73). Strain 130 Δ*pyk* was generated by spot transformation (74) with an overlap extension PCR product, replacing the *pyk* ORF with a spectinomycin resistance cassette using the following primers: Pyk Upstream F-CCGAATACGGCGACTTTACC, Pyk-SacI-Omega F-CAAAATCGTCGCCACCCTTGGAGCTCTGCCCGTTCCATACAGAAGC, Pyk upstream R-GCTTCTGTATGGAACGGGCAGAGCTCCAAGGGTGGCGACGATTTTG, Pyk downstream F-GCTCACAGCCAAACTATCAGGTGAGCTCCAGACGGAGTATCCCGAAGC, Pyk-sacI-Omega R-GCTTCGGGATACTCCGTCTGGAGCTCACCTGATAGTTTGGCTGTGAGC, Pyk downstream R-ACTGTGTGCCGAAGTGGTAG. Mutation was confirmed by sequencing and PCR. Strain 130 Δ*pyk* was genetically complemented by cloning the *pyk* ORF into the pKH35 complementation plasmid (75) using primers SmaI-pyk-F-ACCCCGGGCCGCAAAACACCCGATTGAG and SacI-Pyk-R-AAGAGCTCCAGGGCGGATTATTTGACGC. The allele was incorporated between *lctP* and *aspC* by homologous recombination in 130 Δ*pyk* by spot transformation (74) and selection using chloramphenicol (0.4 µg/mL). This complemented strain is deemed Δ*pyk* :: *pyk^+^*. In Δ*pyk* :: *pyk^+^*, Pyk expression is induced by growing Gc in the presence of 1 mM isopropyl-β-d-thiogalactopyranoside (IPTG).

WT Gc were grown on Gonococcal Medium Base (GCB, Difco) plus Kellogg’s supplements at 37°C with 5% CO_2_ (76, 77). Δ*pyk* strains were grown on GCB plus Kellogg’s supplements with glucose replaced by pyruvate (36 mM) as in reference (78). For preparation of mid-logarithmic phase bacteria, Gc were grown in liquid medium (GCBL) or carbon-matched GCBL containing pyruvate (45 mM) as the sole carbon source, where appropriate, for successive rounds of dilution, and enriched for piliation, as previously described (79). Spectinomycin was used for the selection of the *pyk* mutation at 80 µg/mL.

### Growth curves

Gc in mid-logarithmic phase were pelleted, resuspended in the indicated media, and diluted to ~5*10^7^ CFU/mL in 6 mL of media in 15-mL conical tubes (Sarstedt). The bacterial suspension was incubated with rotation at 37°C. Bacterial growth was measured by OD_550_ and CFU enumeration at specific time points. CFUs are presented relative to 0 hour (100%). Gc was grown in GCBL, HyClone RPMI 1640 media without glutamine (Catalog no. SH30096.FS) (Cytivia), or carbon-matched Morse’s defined media (MDM) containing either glucose (27 mM), lactate (54 mM), or pyruvate (54 mM) (80). Doubling times were calculated from best-fit logistic curves generated with GrowthCurver (69) for the lag and exponential phases of each growth curve for at least three experimental replicates and averaged. Significant differences for growth over time were determined by one-tailed *t*-test in GraphPad Prism v9.

### Gc-PMN co-culture

PMNs were isolated from venous blood as previously described and used within 2 hours of isolation (79). Subjects gave informed consent in accordance with an approved protocol by the University of Virginia Institutional Review Board for Health Sciences Research (no. 13909). Synchronized Gc infection of PMNs in suspension was conducted as previously described (81). PMNs were resuspended in RPMI (Cytivia) containing 10% heat-inactivated FBS (Gibco) at 1 × 10^6^ PMN/mL, and Gc was added to each tube at a multiplicity of infection of 10. One millimolar of IPTG was added where indicated to induce the expression of *pyk*. Six milliliters of the suspension was incubated in 15-mL conical tubes with rotation at 37°C. Bacterial CFUs were enumerated at specified time points and expressed relative to the CFU at 0 hour (100%). Data are expressed as the mean ± SEM of at least three replicate experiments. Significant differences were determined by two-way analysis of variance (ANOVA) with Holm–Sidak correction for multiple comparisons in GraphPad Prism v9.

## Data Availability

Python and R code/packages/scripts, used to perform transcriptomics data analyses and machine learning and to generate figures, are available on GitHub. All RNA-seq data are available in the GEO database under the accession no. GSE123434 (47).

## SUPPLEMENTAL MATERIAL

Data Set S1 msystems.01265-22-s0001.xlsx Annotations on the curation of reactions, metabolites, and genes of iNgo_557.

Data Set S2 msystems.01265-22-s0002.xlsx An Excel file containing calculations to determine biomass components and stoichiometries, and molecular weights using data from the Nmb_iTM560 modified with Gc-specific data.

Data Set S3 msystems.01265-22-s0003.xlsx An Excel file containing the iNgo_557 model reactions, stoichiometries, metabolites, and genes.

Data Set S4 msystems.01265-22-s0004.xlsx *In silico* formulations for GCBL, MDM, and RPMI.

Data Set S5 msystems.01265-22-s0005.xlsx Essential gene predictions for iNgo_557 on simulated GCBL.

Data Set S6 msystems.01265-22-s0006.xlsx Raw transcriptomics read counts for Gc with PMNs and Gc without PMNs.

Data Set S7 msystems.01265-22-s0007.xlsx Essential gene predictions with contextualized models of iNgo_557 for Gc without PMNs and Gc with PMNs.

Supplemental Material msystems.01265-22-s0008.docx Figures S1 to S8; Tables S1 and S2.

ASM does not own the copyrights to Supplemental Material that may be linked to, or accessed through, an article. The authors have granted ASM a non-exclusive, world-wide license to publish the Supplemental Material files. Please contact the corresponding author directly for reuse.

## References

[1] Palmer A , Criss AK . 2018. Gonococcal defenses against antimicrobial activities of neutrophils. Trends Microbiol 26:1022–1034. doi:10.1016/j.tim.2018.07.003 30115561 PMC6251743

[2] Quillin SJ , Seifert HS . 2018. Neisseria gonorrhoeae host adaptation and pathogenesis. Nat Rev Microbiol 16:226–240. doi:10.1038/nrmicro.2017.169 29430011 PMC6329377

[3] Morse SA , Bartenstein L . 1974. Factors affecting autolysis of Neisseria gonorrhoeae. Proc Soc Exp Biol Med 145:1418–1421. doi:10.3181/00379727-145-38025 4208046

[4] Atack JM , Ibranovic I , Ong C-L , Djoko KY , Chen NH , Vanden Hoven R , Jennings MP , Edwards JL , McEwan AG . 2014. A role for lactate dehydrogenases in the survival of Neisseria gonorrhoeae in human polymorphonuclear leukocytes and cervical epithelial cells. J Infect Dis 210:1311–1318. doi:10.1093/infdis/jiu230 24737798 PMC4215069

[5] Chen NH , Ong C-L , O’sullivan J , Ibranovic I , Davey K , Edwards JL , McEwan AG . 2020. Two distinct L-lactate dehydrogenases play a role in the survival of Neisseria gonorrhoeae in cervical epithelial cells. J Infect Dis 221:449–453. doi:10.1093/infdis/jiz468 31541571 PMC7530546

[6] Cornelissen CN . 2018. Subversion of nutritional immunity by the pathogenic Neisseriae. Pathog Dis 76:ftx112. doi:10.1093/femspd/ftx112 29045638 PMC6251569

[7] Muenzner P , Hauck CR . 2020. Neisseria gonorrhoeae blocks epithelial exfoliation by nitric-oxide-mediated metabolic cross talk to promote colonization in mice. Cell Host Microbe 27:793–808. doi:10.1016/j.chom.2020.03.010 32289262

[8] Stoudenmire JL , Greenawalt AN , Cornelissen CN . 2022. Stealthy microbes: how Neisseria gonorrhoeae hijacks bulwarked iron during infection. Front Cell Infect Microbiol 12:1017348. doi:10.3389/fcimb.2022.1017348 36189345 PMC9519893

[9] Branch AH , Stoudenmire JL , Seib KL , Cornelissen CN . 2022. Acclimation to nutritional immunity and metal intoxication requires zinc, manganese, and copper homeostasis in the pathogenic Neisseriae. Front Cell Infect Microbiol 12:909888. doi:10.3389/fcimb.2022.909888 35846739 PMC9280163

[10] Waltmann A , Duncan JA , Pier GB , Cywes-Bentley C , Cohen MS , Hobbs MM . 2022. Experimental urethral infection with Neisseria gonorrhoeae. Curr Top Microbiol Immunol. doi:10.1007/82_2021_250 PMC9441470 35246736

[11] Cornelissen CN , Kelley M , Hobbs MM , Anderson JE , Cannon JG , Cohen MS , Sparling PF . 1998. The transferrin receptor expressed by gonococcal strain FA1090 is required for the experimental infection of human male volunteers. Mol Microbiol 27:611–616. doi:10.1046/j.1365-2958.1998.00710.x 9489672

[12] Zimmermann J , Kaleta C , Waschina S . 2021. Gapseq: informed prediction of bacterial metabolic pathways and reconstruction of accurate metabolic models. Genome Biol 22:81. doi:10.1186/s13059-021-02295-1 33691770 PMC7949252

[13] Tamasco G , Kumar M , Zengler K , Silva-Rocha R , da Silva RR . 2022. ChiMera: an easy to use pipeline for bacterial genome based metabolic network reconstruction, evaluation and visualization. BMC Bioinformatics 23:512. doi:10.1186/s12859-022-05056-4 36451100 PMC9710178

[14] Karlsen E , Schulz C , Almaas E . 2018. Automated generation of genome-scale metabolic draft reconstructions based on KEGG. BMC Bioinformatics 19:467. doi:10.1186/s12859-018-2472-z 30514205 PMC6280343

[15] Moutinho TJ , Neubert BC , Jenior ML , Papin JA . 2022. Quantifying cumulative phenotypic and genomic evidence for procedural generation of metabolic network reconstructions. PLoS Comput Biol 18:e1009341. doi:10.1371/journal.pcbi.1009341 35130271 PMC8853471

[16] Zhang C , Hua Q . 2015. Applications of genome-scale metabolic models in biotechnology and systems medicine. Front Physiol 6:413. doi:10.3389/fphys.2015.00413 26779040 PMC4703781

[17] Jenior ML , Leslie JL , Powers DA , Garrett EM , Walker KA , Dickenson ME , Petri WA , Tamayo R , Papin JA . 2021. Novel drivers of virulence in Clostridioides difficile identified via context-specific metabolic network analysis. mSystems 6:e0091921. doi:10.1128/mSystems.00919-21 34609164 PMC8547418

[18] Sertbas M , Ulgen KO . 2020. Genome-scale metabolic modeling for unraveling molecular mechanisms of high threat pathogens. Front Cell Dev Biol 8:566702. doi:10.3389/fcell.2020.566702 33251208 PMC7673413

[19] Payne DD , Renz A , Dunphy LJ , Lewis T , Dräger A , Papin JA . 2021. An updated genome-scale metabolic network reconstruction of Pseudomonas aeruginosa PA14 to characterize mucin-driven shifts in bacterial metabolism. NPJ Syst Biol Appl 7:37. doi:10.1038/s41540-021-00198-2 34625561 PMC8501023

[20] Hartman HB , Fell DA , Rossell S , Jensen PR , Woodward MJ , Thorndahl L , Jelsbak L , Olsen JE , Raghunathan A , Daefler S , Poolman MG . 2014. Identification of potential drug targets in Salmonella enterica sv. Typhimurium using metabolic Modelling and experimental validation. Microbiology 160:1252–1266. doi:10.1099/mic.0.076091-0 24777662

[21] Mazharul Islam M , Thomas VC , Van Beek M , Ahn JS , Alqarzaee AA , Zhou C , Fey PD , Bayles KW , Saha R . 2020. An integrated computational and experimental study to investigate Staphylococcus aureus metabolism. NPJ Syst Biol Appl 6:3. doi:10.1038/s41540-019-0122-3 32001720 PMC6992624

[22] Kavvas ES , Seif Y , Yurkovich JT , Norsigian C , Poudel S , Greenwald WW , Ghatak S , Palsson BO , Monk JM . 2018. Updated and standardized genome-scale reconstruction of Mycobacterium tuberculosis H37Rv, IEk1011, simulates flux states indicative of physiological conditions. BMC Syst Biol 12:25. doi:10.1186/s12918-018-0557-y 29499714 PMC5834885

[23] Mendum TA , Newcombe J , Mannan AA , Kierzek AM , McFadden J . 2011. Interrogation of global mutagenesis data with a genome scale model of Neisseria meningitidis to assess gene fitness in vitro and in sera. Genome Biol 12:R127. doi:10.1186/gb-2011-12-12-r127 22208880 PMC3334622

[24] Derkaoui M , Antunes A , Nait Abdallah J , Poncet S , Mazé A , Ma Pham QM , Mokhtari A , Deghmane A-E , Joyet P , Taha M-K , Deutscher J . 2016. Transport and catabolism of carbohydrates by Neisseria Meningitidis. J Mol Microbiol Biotechnol 26:320–332. doi:10.1159/000447093 27454890

[25] Gu C , Kim GB , Kim WJ , Kim HU , Lee SY . 2019. Current status and applications of genome-scale metabolic models. Genome Biol 20:121. doi:10.1186/s13059-019-1730-3 31196170 PMC6567666

[26] King ZA , Lu J , Dräger A , Miller P , Federowicz S , Lerman JA , Ebrahim A , Palsson BO , Lewis NE . 2016. BiGG models: a platform for integrating, standardizing and sharing genome-scale models. Nucleic Acids Res 44:D515–D522. doi:10.1093/nar/gkv1049 26476456 PMC4702785

[27] Norsigian CJ , Fang X , Seif Y , Monk JM , Palsson BO . 2020. A workflow for generating multi-strain genome-scale metabolic models of prokaryotes. Nat Protoc 15:1–14. doi:10.1038/s41596-019-0254-3 31863076 PMC7017905

[28] Keating SM , Waltemath D , König M , Zhang F , Dräger A , Chaouiya C , Bergmann FT , Finney A , Gillespie CS , Helikar T , Hoops S , Malik-Sheriff RS , Moodie SL , Moraru II , Myers CJ , Naldi A , Olivier BG , Sahle S , Schaff JC , Smith LP , Swat MJ , Thieffry D , Watanabe L , Wilkinson DJ , Blinov ML , Begley K , Faeder JR , Gómez HF , Hamm TM , Inagaki Y , Liebermeister W , Lister AL , Lucio D , Mjolsness E , Proctor CJ , Raman K , Rodriguez N , Shaffer CA , Shapiro BE , Stelling J , Swainston N , Tanimura N , Wagner J , Meier-Schellersheim M , Sauro HM , Palsson B , Bolouri H , Kitano H , Funahashi A , Hermjakob H , Doyle JC , Hucka M , SBML Level 3 Community members . 2020. SBML level 3: an extensible format for the exchange and reuse of biological models. Mol Syst Biol 16:e9110. doi:10.15252/msb.20199110 32845085 PMC8411907

[29] Devoid S , Overbeek R , DeJongh M , Vonstein V , Best AA , Henry C . 2013. Automated genome annotation and metabolic model reconstruction in the SEED and model SEED. Methods Mol Biol 985:17–45. doi:10.1007/978-1-62703-299-5_2 23417797

[30] Wattam AR , Davis JJ , Assaf R , Boisvert S , Brettin T , Bun C , Conrad N , Dietrich EM , Disz T , Gabbard JL , Gerdes S , Henry CS , Kenyon RW , Machi D , Mao C , Nordberg EK , Olsen GJ , Murphy-Olson DE , Olson R , Overbeek R , Parrello B , Pusch GD , Shukla M , Vonstein V , Warren A , Xia F , Yoo H , Stevens RL . 2017. Improvements to PATRIC, the all-bacterial bioinformatics database and analysis resource center. Nucleic Acids Res 45:D535–D542. doi:10.1093/nar/gkw1017 27899627 PMC5210524

[31] Moretti S , Tran VDT , Mehl F , Ibberson M , Pagni M . 2021. MetaNetX/MNXref: unified namespace for metabolites and biochemical reactions in the context of metabolic models. Nucleic Acids Res 49:D570–D574. doi:10.1093/nar/gkaa992 33156326 PMC7778905

[32] Caspi R , Billington R , Keseler IM , Kothari A , Krummenacker M , Midford PE , Ong WK , Paley S , Subhraveti P , Karp PD . 2020. The MetaCyc database of metabolic pathways and enzymes - a 2019 update. Nucleic Acids Res 48:D445–D453. doi:10.1093/nar/gkz862 31586394 PMC6943030

[33] Jolley KA , Bray JE , Maiden MCJ . 2018. Open-access bacterial population genomics: BIGSdb software, the PubMLST.org website and their applications. Wellcome Open Res 3:124. doi:10.12688/wellcomeopenres.14826.1 30345391 PMC6192448

[34] Kanehisa M , Sato Y , Kawashima M . 2022. KEGG mapping tools for Uncovering hidden features in biological data. Protein Sci 31:47–53. doi:10.1002/pro.4172 34423492 PMC8740838

[35] Beste DJV , Hooper T , Stewart G , Bonde B , Avignone-Rossa C , Bushell ME , Wheeler P , Klamt S , Kierzek AM , McFadden J . 2007. GSMN-TB: a web-based genome-scale network model of mycobacterium tuberculosis metabolism. Genome Biol 8:R89. doi:10.1186/gb-2007-8-5-r89 17521419 PMC1929162

[36] Xavier JC , Patil KR , Rocha I . 2017. Integration of biomass formulations of genome-scale metabolic models with experimental data reveals universally essential cofactors in Prokaryotes. Metab Eng 39:200–208. doi:10.1016/j.ymben.2016.12.002 27939572 PMC5249239

[37] Machado D , Andrejev S , Tramontano M , Patil KR . 2018. Fast automated reconstruction of genome-scale metabolic models for microbial species and communities. Nucleic Acids Res 46:7542–7553. doi:10.1093/nar/gky537 30192979 PMC6125623

[38] Lieven C , Beber ME , Olivier BG , Bergmann FT , Ataman M , Babaei P , Bartell JA , Blank LM , Chauhan S , Correia K , Diener C , Dräger A , Ebert BE , Edirisinghe JN , Faria JP , Feist AM , Fengos G , Fleming RMT , García-Jiménez B , Hatzimanikatis V , van Helvoirt W , Henry CS , Hermjakob H , Herrgård MJ , Kaafarani A , Kim HU , King Z , Klamt S , Klipp E , Koehorst JJ , König M , Lakshmanan M , Lee D-Y , Lee SY , Lee S , Lewis NE , Liu F , Ma H , Machado D , Mahadevan R , Maia P , Mardinoglu A , Medlock GL , Monk JM , Nielsen J , Nielsen LK , Nogales J , Nookaew I , Palsson BO , Papin JA , Patil KR , Poolman M , Price ND , Resendis-Antonio O , Richelle A , Rocha I , Sánchez BJ , Schaap PJ , Sheriff RSM , Shoaie S , Sonnenschein N , Teusink B , Vilaça P , Vik JO , Wodke JAH , Xavier JC , Yuan Q , Zakhartsev M , Zhang C . 2020. MEMOTE for standardized genome-scale metabolic model testing. Nat Biotechnol 38:272–276. doi:10.1038/s41587-020-0477-4 32123384 PMC7082222

[39] Orth JD , Thiele I , Palsson BØ . 2010. What is flux balance analysis? Nat Biotechnol 28:245–248. doi:10.1038/nbt.1614 20212490 PMC3108565

[40] Marinos G , Kaleta C , Waschina S . 2020. Defining the nutritional input for genome-scale metabolic models: a roadmap. PLoS One 15:e0236890. doi:10.1371/journal.pone.0236890 32797084 PMC7428157

[41] Steiner T , Zachary M , Bauer S , Müller MJ , Krischke M , Radziej S , Klepsch M , Huettel B , Eisenreich W , Rudel T , Beier D , Storz G . 2023. Central role of sibling small RNAs NgncR_162 and NgncR_163 in main metabolic pathways of Neisseria gonorrhoeae. mBio 14:e0309322. doi:10.1128/mbio.03093-22 36598194 PMC9973317

[42] Remmele CW , Xian Y , Albrecht M , Faulstich M , Fraunholz M , Heinrichs E , Dittrich MT , Müller T , Reinhardt R , Rudel T . 2014. Transcriptional landscape and essential genes of Neisseria gonorrhoeae. Nucleic Acids Res 42:10579–10595. doi:10.1093/nar/gku762 25143534 PMC4176332

[43] Blazier AS , Papin JA . 2019. Reconciling high-throughput gene essentiality data with metabolic network reconstructions. PLoS Comput Biol 15:e1006507. doi:10.1371/journal.pcbi.1006507 30973869 PMC6478342

[44] Rosconi F , Rudmann E , Li J , Surujon D , Anthony J , Frank M , Jones DS , Rock C , Rosch JW , Johnston CD , van Opijnen T . 2022. A bacterial pan-genome makes gene essentiality strain-dependent and evolvable. Nat Microbiol 7:1580–1592. doi:10.1038/s41564-022-01208-7 36097170 PMC9519441

[45] Blazier AS , Papin JA . 2012. Integration of expression data in genome-scale metabolic network reconstructions. Front Physiol 3:299. doi:10.3389/fphys.2012.00299 22934050 PMC3429070

[46] Smith AB , Jenior ML , Keenan O , Hart JL , Specker J , Abbas A , Rangel PC , Di C , Green J , Bustin KA , Gaddy JA , Nicholson MR , Laut C , Kelly BJ , Matthews ML , Evans DR , Van Tyne D , Furth EE , Papin JA , Bushman FD , Erlichman J , Baldassano RN , Silverman MA , Dunny GM , Prentice BM , Skaar EP , Zackular JP . 2022. Enterococci enhance Clostridioides difficile pathogenesis. Nature 611:780–786. doi:10.1038/s41586-022-05438-x 36385534 PMC9691601

[47] Edwards VL , Potter AD , D’Mello A , Gray MC , Shetty AC , Zhao X , Hill KM , Ragland SA , Criss AK , Tettelin H . 2022. Dual species transcriptomics reveals core metabolic and immunologic processes in the interaction between primary human neutrophils and Neisseria gonorrhoeae strains . Genomics. doi:10.1101/2022.02.28.482360 PMC11257400 38976720

[48] Ball LM , Criss AK . 2013. Constitutively Opa-expressing and Opa-deficient Neisseria gonorrhoeae strains differentially stimulate and survive exposure to human neutrophils. J Bacteriol 195:2982–2990. doi:10.1128/JB.00171-13 23625842 PMC3697530

[49] Janitza S , Strobl C , Boulesteix AL . 2013. An AUC-based permutation variable importance measure for random forests. BMC Bioinformatics 14:119. doi:10.1186/1471-2105-14-119 23560875 PMC3626572

[50] Breiman L . 2001. Random forests. Mach Learn 45:5–32. doi:10.1023/A:1010933404324

[51] Britigan BE , Klapper D , Svendsen T , Cohen MS . 1988. Phagocyte-derived lactate stimulates oxygen consumption by Neisseria gonorrhoeae. An unrecognized aspect of the oxygen metabolism of phagocytosis. J Clin Invest 81:318–324. doi:10.1172/JCI113323 3123517 PMC329573

[52] Saini R , Singh S . 2019. Inducible nitric oxide synthase: an asset to neutrophils. J Leukoc Biol 105:49–61. doi:10.1002/JLB.4RU0418-161R 30285282

[53] Green LR , Cole J , Parga EFD , Shaw JG . 2022. Neisseria gonorrhoeae physiology and pathogenesis. Adv Microb Physiol 80:35–83. doi:10.1016/bs.ampbs.2022.01.002 35489793

[54] Basan M , Hui S , Okano H , Zhang Z , Shen Y , Williamson JR , Hwa T . 2015. Overflow metabolism in Escherichia coli results from efficient proteome allocation. Nature 528:99–104. doi:10.1038/nature15765 26632588 PMC4843128

[55] Li G , Lu C-D . 2016. The cryptic dsdA gene encodes a functional D-serine dehydratase in Pseudomonas aeruginosa PAO1. Curr Microbiol 72:788–794. doi:10.1007/s00284-016-1021-0 26957519

[56] Reznik E , Christodoulou D , Goldford JE , Briars E , Sauer U , Segrè D , Noor E . 2017. Genome-scale architecture of small molecule regulatory networks and the fundamental trade-off between regulation and enzymatic activity. Cell Rep 20:2666–2677. doi:10.1016/j.celrep.2017.08.066 28903046 PMC5600504

[57] Potter AD , Butrico CE , Ford CA , Curry JM , Trenary IA , Tummarakota SS , Hendrix AS , Young JD , Cassat JE . 2020. Host nutrient milieu drives an essential role for aspartate biosynthesis during invasive Staphylococcus aureus infection. Proc Natl Acad Sci U S A 117:12394–12401. doi:10.1073/pnas.1922211117 32414924 PMC7275739

[58] Exley RM , Wu H , Shaw J , Schneider MC , Smith H , Jerse AE , Tang CM . 2007. Lactate acquisition promotes successful colonization of the murine genital tract by Neisseria gonorrhoeae. Infect Immun 75:1318–1324. doi:10.1128/IAI.01530-06 17158905 PMC1828543

[59] Leighton MP , Kelly DJ , Williamson MP , Shaw JG . 2001. An NMR and enzyme study of the carbon metabolism of Neisseria meningitidis. Microbiology 147:1473–1482. doi:10.1099/00221287-147-6-1473 11390678

[60] Vincent LR , Kerr SR , Tan Y , Tomberg J , Raterman EL , Dunning Hotopp JC , Unemo M , Nicholas RA , Jerse AE . 2018. In Vivo-selected compensatory mutations restore the fitness cost of mosaic penA Alleles that confer ceftriaxone resistance in Neisseria gonorrhoeae. mBio 9:e01905-17. doi:10.1128/mBio.01905-17 PMC5885032 29615507

[61] Workowski KA , Bachmann LH , Chan PA , Johnston CM , Muzny CA , Park I , Reno H , Zenilman JM , Bolan GA . 2021. Sexually transmitted infections treatment guidelines. MMWR Recomm Rep 70:1–187. doi:10.15585/mmwr.rr7004a1 PMC8344968 34292926

[62] Capasso C , Supuran CT . 2014. Sulfa and trimethoprim-like drugs - antimetabolites acting as carbonic anhydrase, dihydropteroate synthase and dihydrofolate reductase inhibitors. J Enzyme Inhib Med Chem 29:379–387. doi:10.3109/14756366.2013.787422 23627736

[63] Zampieri M , Enke T , Chubukov V , Ricci V , Piddock L , Sauer U . 2017. Metabolic constraints on the evolution of antibiotic resistance. Mol Syst Biol 13:917. doi:10.15252/msb.20167028 28265005 PMC5371735

[64] Smith H , Tang CM , Exley RM . 2007. Effect of host lactate on gonococci and meningococci: new concepts on the role of metabolites in pathogenicity. Infect Immun 75:4190–4198. doi:10.1128/IAI.00117-07 17562766 PMC1951187

[65] Ebrahim A , Lerman JA , Palsson BO , Hyduke DR . 2013. COBRApy: COnstraints-based reconstruction and analysis for python. BMC Syst Biol 7:74. doi:10.1186/1752-0509-7-74 23927696 PMC3751080

[66] Römer M , Eichner J , Dräger A , Wrzodek C , Wrzodek F , Zell A . 2016. ZBIT bioinformatics toolbox: a web-platform for systems biology and expression data analysis. PLoS One 11:e0149263. doi:10.1371/journal.pone.0149263 26882475 PMC4801062

[67] Kanehisa M , Furumichi M , Sato Y , Kawashima M , Ishiguro-Watanabe M . 2022. KEGG for taxonomy-based analysis of pathways and genomes. Nucleic Acids Res. doi:10.1093/nar/gkac963:gkac963 PMC9825424 36300620

[68] Chicco D , Jurman G . 2020. The advantages of the matthews correlation coefficient (MCC) over F1 score and accuracy in binary classification evaluation. BMC Genomics 21:6. doi:10.1186/s12864-019-6413-7 31898477 PMC6941312

[69] Sprouffske K , Wagner A . 2016. Growthcurver: an R package for obtaining interpretable metrics from microbial growth curves. BMC Bioinformatics 17:172. doi:10.1186/s12859-016-1016-7 27094401 PMC4837600

[70] Jenior ML , Moutinho TJ , Dougherty BV , Papin JA , Lewis NE . 2020. Transcriptome-guided parsimonious flux analysis improves predictions with metabolic networks in complex environments. PLoS Comput Biol 16:e1007099. doi:10.1371/journal.pcbi.1007099 32298268 PMC7188308

[71] Dixon P . 2003. VEGAN, a package of R functions for community ecology. Journal of Vegetation Science 14:927–930. doi:10.1111/j.1654-1103.2003.tb02228.x

[72] King ZA , Dräger A , Ebrahim A , Sonnenschein N , Lewis NE , Palsson BO , Gardner PP . 2015. Escher: a web application for building, sharing, and embedding data-rich visualizations of biological pathways. PLoS Comput Biol 11:e1004321. doi:10.1371/journal.pcbi.1004321 26313928 PMC4552468

[73] Cahoon LA , Seifert HS . 2009. An alternative DNA structure is necessary for pilin antigenic variation in Neisseria gonorrhoeae. Science 325:764–767. doi:10.1126/science.1175653 19661435 PMC2803317

[74] Dillard JP . 2011. Genetic manipulation of Neisseria gonorrhoeae. Curr Protoc Microbiol Chapter 4:Unit4A.2. doi:10.1002/9780471729259.mc04a02s23 PMC4549065 22045584

[75] Ramsey ME , Hackett KT , Kotha C , Dillard JP . 2012. New complementation constructs for inducible and constitutive gene expression in Neisseria gonorrhoeae and Neisseria meningitidis. Appl Environ Microbiol 78:3068–3078. doi:10.1128/AEM.07871-11 22327577 PMC3346468

[76] Kellogg DS , Peacock WL , Deacon WE , Brown L , Pirkle CI . 1963. Neisseria gonorrhoeae I: virulence genetically linked to clonal variation. J Bacteriol 85:1274–1279. doi:10.1128/jb.85.6.1274-1279.1963 14047217 PMC278328

[77] Ragland SA , Criss AK . 2019. Protocols to interrogate the interactions between Neisseria gonorrhoeae and primary human neutrophils. Methods Mol Biol 1997:319–345. doi:10.1007/978-1-4939-9496-0_19 31119632 PMC6731993

[78] Williams JM , Chen GC , Zhu L , Rest RF . 1998. Using the yeast two-hybrid system to identify human epithelial cell proteins that bind gonococcal Opa proteins: intracellular gonococci bind pyruvate kinase via their Opa proteins and require host pyruvate for growth. Mol Microbiol 27:171–186. doi:10.1046/j.1365-2958.1998.00670.x 9466265

[79] Criss AK , Seifert HS . 2008. Neisseria gonorrhoeae suppresses the oxidative burst of human polymorphonuclear leukocytes. Cell Microbiol 10:2257–2270. doi:10.1111/j.1462-5822.2008.01205.x 18684112 PMC2692872

[80] Morse SA , Bartenstein L . 1980. Purine metabolism in Neisseria gonorrhoeae: the requirement for hypoxanthine. Can J Microbiol 26:13–20. doi:10.1139/m80-003 6773641

[81] Rest RF , Speert DP . 1994. Measurement of nonopsonic phagocytic killing by human and mouse phagocytes. Methods Enzymol 236:91–108. doi:10.1016/0076-6879(94)36010-3 7968642

