# Supplementary material for: Transcriptome-guided metabolic network analysis reveals rearrangements of carbon flux distribution in Neisseria gonorrhoeae during neutrophil co-culture
Source: mSystems. 2023 Jun 30;8(4):e01265-22. doi: 10.1128/msystems.01265-22 (PMC10470122; doi:10.1128/msystems.01265-22)
Supplement: Supplemental Material — Figures S1 to S8; Tables S1 and S2. [file msystems.01265-22-s0008.docx]

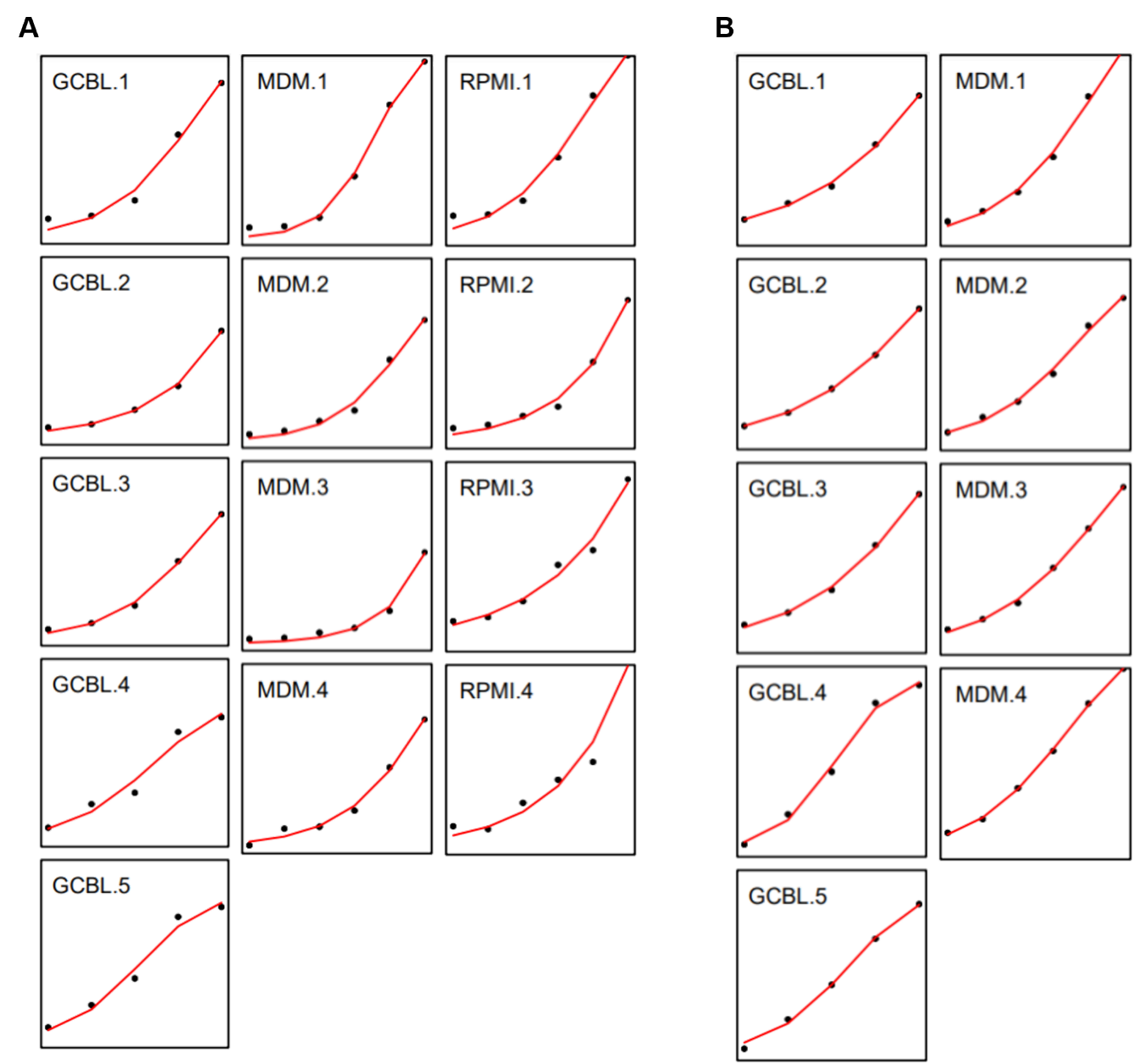


**Fig S1: Best fit logistic curves generated with GrowthCurver were used to calculate experimental doubling time for Gc grown in GCBL, MDM, and RPMI.** Log phase WT Gc was backdiluted into GCBL, MDM, or RPMI. Growth over 5 hours was monitored by (A) enumeration of CFU/ml or (B) optical density at 550 nm. Optical density for Gc grown in RPMI was not reported due to phenol red indicators in the media. n = 4-5 biological replicates.


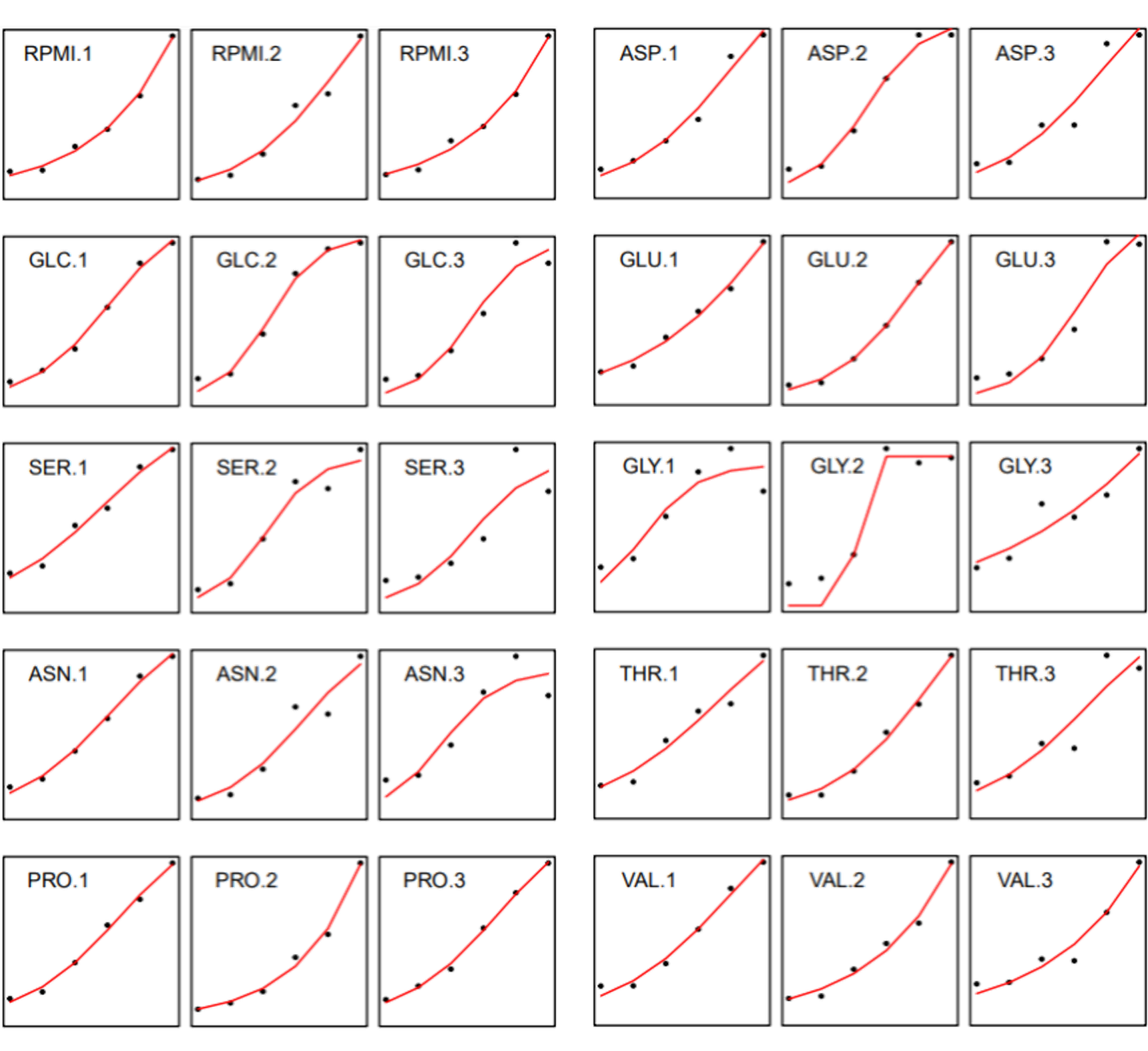


**Fig S2: Best fit logistic curves generated with GrowthCurver were used to calculate experimental doubling time of Gc grown in RPMI supplemented with potential limiting metabolites.** Log phase WT Gc was backdiluted into RPMI supplemented with 5X the standard concentration of metabolites indicated in **Fig. 3**. Glucose (GLC), serine (SER), asparagine (ASN), proline (PRO), aspartate (ASP), glutamate (GLU), and glycine (GLY) were predicted to be growth limiting; threonine (THR) and valine (VAL) were not predicted to be growth limiting. Growth over 5 hours was monitored by enumeration of CFU/ml. n = 3 biological replicates per condition.


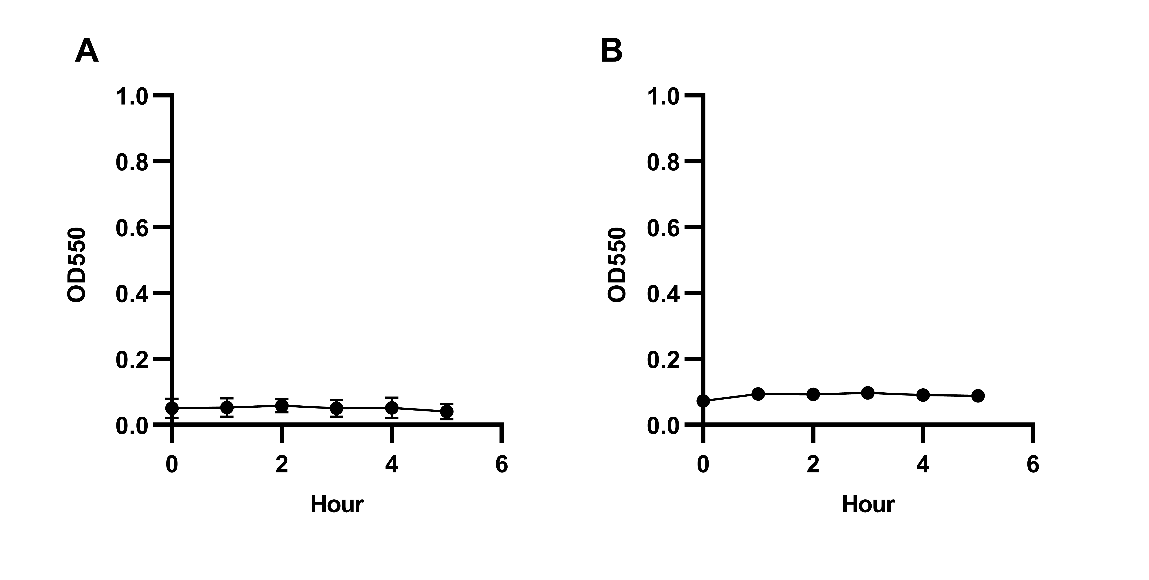


**Fig S3:** ***N. gonorrhoeae* requires glucose, pyruvate, or lactate as a carbon source for growth.** Log phase WT Gc was backdiluted into MDM containing (A) no dedicated carbon source (no glucose, lactate, or pyruvate) or (B) with 1% Casamino acids added as the carbon source. Growth was monitored by optical density at 550 nm over 5 hours. (A) n = 3 biological replicates. Symbols represent the mean. Error bars represent SEM. (B) n = 1 biological replicate.


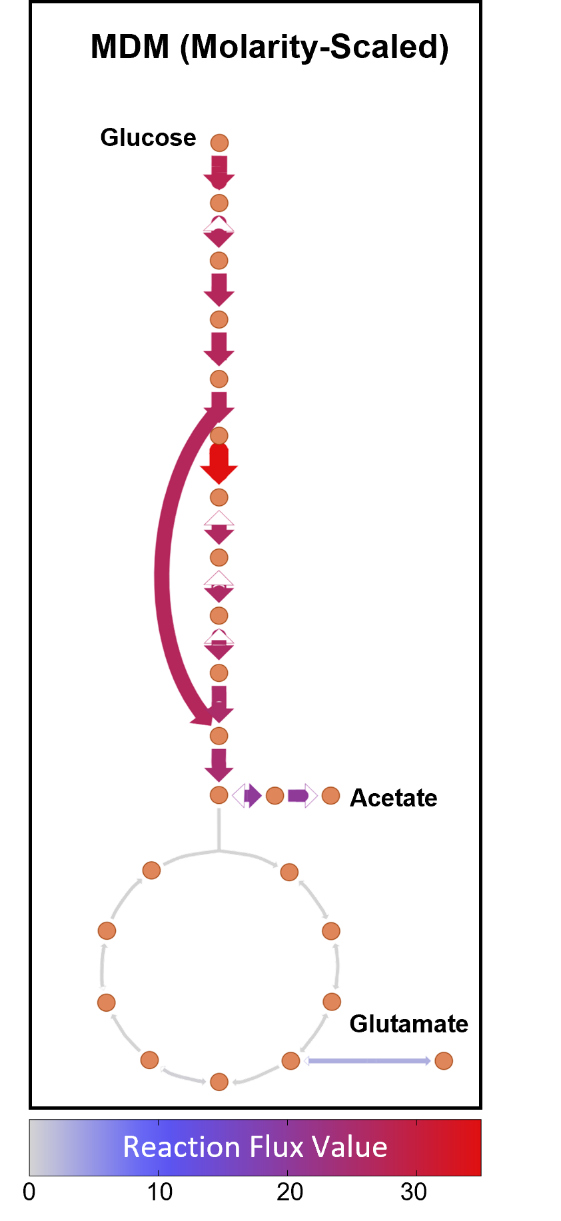


**Fig S4: Visualization of flux balance analysis for central carbon metabolism in iNgo_557 during growth on MDM.** Orange circles indicate metabolites. Relevant imported and exported metabolites are indicated in bold. Arrows indicate reactions. The intensity of coloration and the arrow size indicate the degree of flux through reactions. Schematics were generated with Escher.


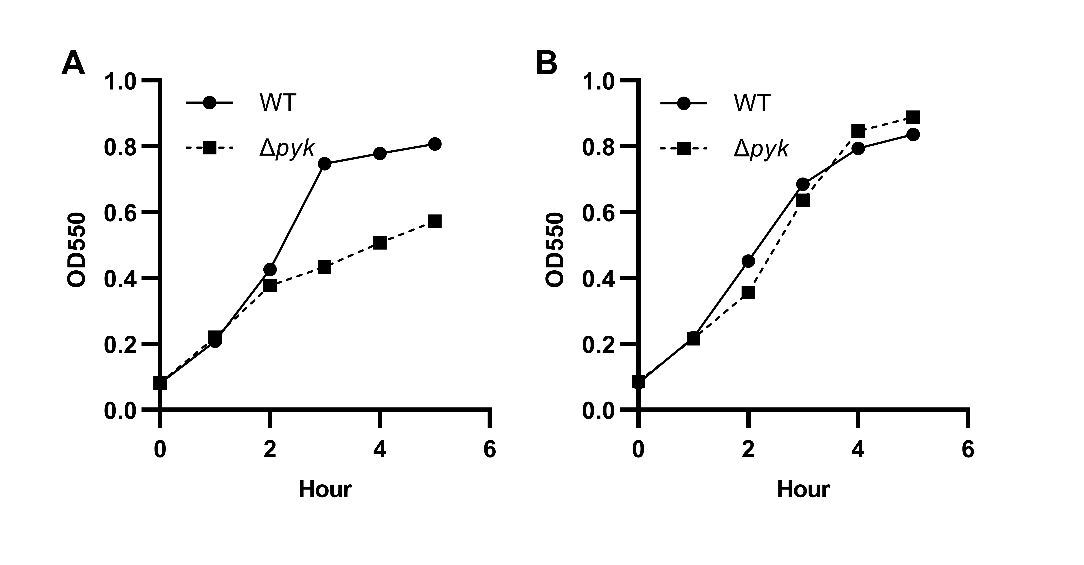


**Fig S5: Growth dynamics of WT and Δ*pyk N. gonorrhoeae* in GCBL with different carbon sources.** Log phase WT Gc and an isogenic Δ*pyk* mutant were backdiluted into GCBL containing (A) 22 mM glucose or (B) 45 mM pyruvate as the primary carbon source and grown for 5 hours. Growth was monitored by optical density at 550 nm for n = 1 biological replicate.


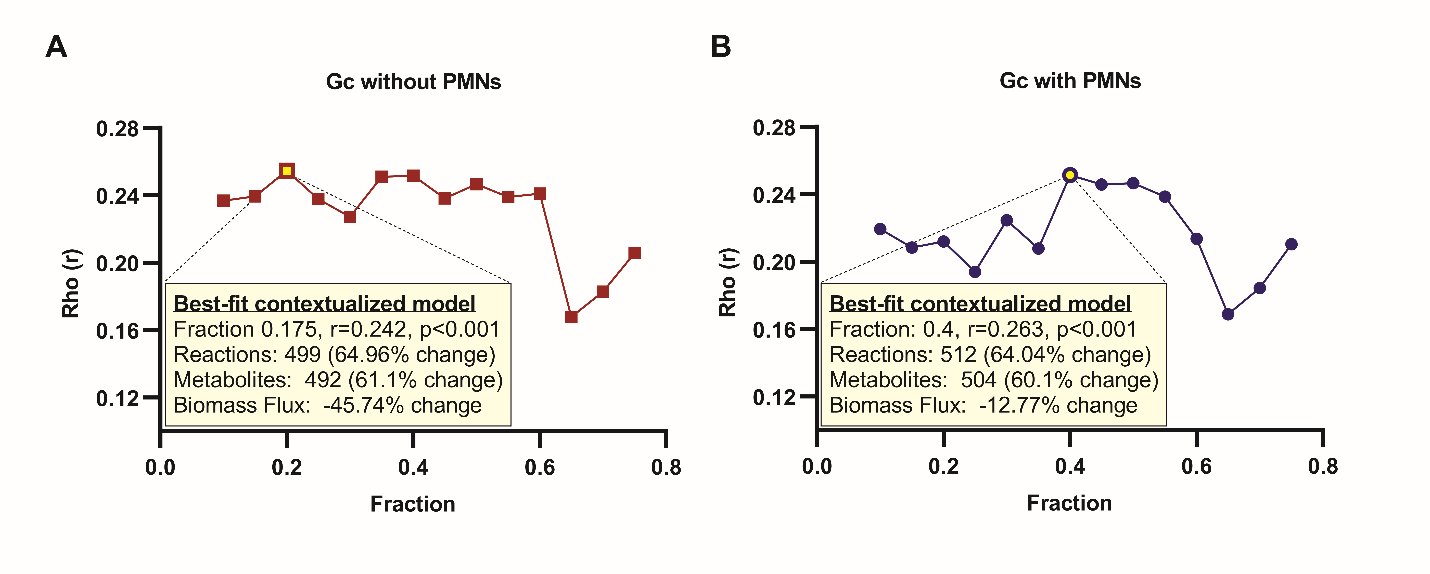


**Fig S6: Application of RIPTiDe to iNgo_557 generates best-fit contextualized models integrated with transcriptional data.** RIPTiDe was used to produce contextualized models for Gc grown in the presence or absence of PMNs. Biomass flux of iNgo_557 was iteratively constrained to 10-80% of the maximum (Fraction: 0.1-0.8) in 5% increments. RIPTiDe then assigns linear coefficients to model reactions based on corresponding transcript abundance. Flux balance analysis is used to identify unused (zero flux) reactions which are removed (i.e. “pruned”) from the model, along with unused genes and metabolites. The Spearman correlation coefficient (Rho, r) for each fraction is calculated through correlation of transcript abundance and median flux value for each corresponding reaction, and the fraction with the highest Rho (yellow) is chosen as the model that best aligns with transcriptomic data. Metrics for the best-fit contextualized model are boxed.


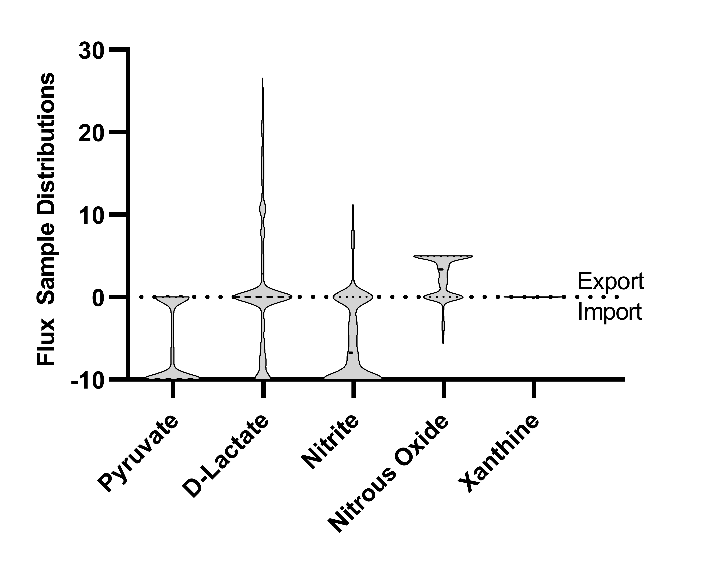


**Fig S7. Metabolic activity predictions for exchange reactions unique to Gc co-cultured with PMNs.** The distribution of flux samples for uniquely active exchange reactions in the Gc cultured with PMNs context-specific model. Negative exchanges indicate flux into the model (“import”). Positive exchanges indicate flux out of the model (“export”). The median of all exchange reactions is near 0.


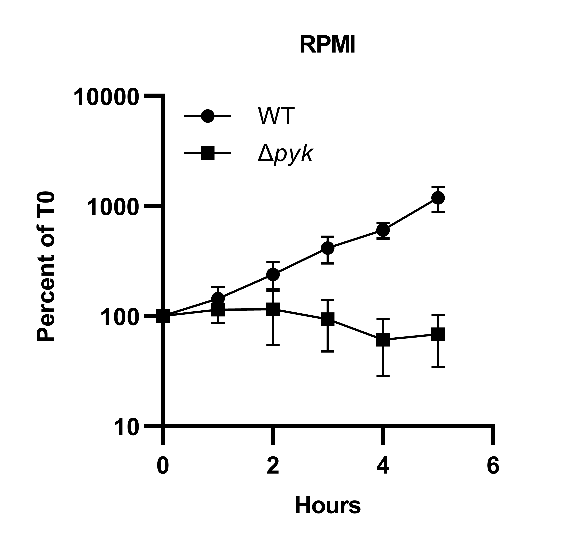


**Fig S8:** **A *N. gonorrhoeae pyk* mutant does not grow in RPMI containing glucose as sole carbon source.** Log phase WT Gc and an isogenic Δ*pyk* mutant were backdiluted into RPMI. Growth over 5 hours was monitored by enumeration of CFU/ml reported as percent of CFU measured at 0 hours (100%). Symbols represent the mean. Error bars represent SEM. n=3 biological replicates.

**Table S1: Concentrations of nutrients predicted to be limiting for Gc growth in RPMI.**

|  | **Standard Concentration (mM)** | **5X Concentration (mM)** |
| --- | --- | --- |
| ASN | 0.378 | 1.892 |
| ASP | 0.150 | 0.752 |
| Glucose | 11.101 | 55.506 |
| GLU | 0.136 | 0.680 |
| GLY | 0.133 | 0.666 |
| PRO | 0.174 | 0.869 |
| SER | 0.286 | 1.428 |
| THR | 0.168 | 0.840 |
| VAL | 0.171 | 0.854 |

|  | **RPMI  (Equally-Scaled)** | | **RPMI  (Molarity-Scaled)** | |
| --- | --- | --- | --- | --- |
|  | **Predicted  Doubling Time** | **Ratio of 5X/Original^1^** | **Predicted  Doubling Time** | **Ratio of 5X/Original^1^** |
| unmodified RPMI | 30 | - | 146 | - |
| Glucose | 23 | 0.76 | 146 | 1 |
| SER | 25 | 0.83 | 88 | 0.60 |
| ASN | 30 | 1 | 56 | 0.38 |
| PRO | 30 | 1 | 103 | 0.71 |
| ASP | 30 | 1 | 107 | 0.73 |
| GLU | 30 | 1 | 110 | 0.75 |
| GLY | 30 | 1 | 110 | 0.75 |
| THR | 30 | 1 | 146 | 1 |
| VAL | 30 | 1 | 146 | 1 |

**Table S2: Growth predictions for *N. gonorrhoeae* in RPMI in equally-scaled or molarity-scaled models when selected metabolites are added at five-fold the original constraint.**

^1^ Increase in predicted doubling time when the indicated metabolite is increased by five-fold (5x), expressed relative to doubling time in unmodified RPMI.
